# Involvement of an octose ketoreductase and two acyltransferases in the biosynthesis of paulomycins

**DOI:** 10.1038/srep21180

**Published:** 2016-02-15

**Authors:** Jine Li, Min Wang, Yong Ding, Yue Tang, Zhiguo Zhang, Yihua Chen

**Affiliations:** 1 State Key Laboratory of Microbial Resources, Institute of Microbiology, Chinese Academy of Sciences, Beijing 100101, China; 2 University of Chinese Academy of Sciences, Beijing, 110039, China; 3 QiLu University of Technology, Jinan, Shandong Province, 250353,China

## Abstract

C-4 hydroxyethyl branched octoses have been observed in polysaccharides of several genera of gram negative bacteria and in various antibiotics produced by gram positive bacteria. The C-4 hydroxyethyl branch was proposed to be converted from C-4 acetyl branch by an uncharacterized ketoreduction step. Paulomycins (PAUs) are glycosylated antibiotics with potent inhibitory activity against gram positive bacteria and are structurally defined by its unique C-4′ hydroxyethyl branched paulomycose moiety. A novel aldo-keto-reductase, Pau7 was characterized as the enzyme catalyzing the stereospecific ketoreduction of 7′-keto of PAU E (1) to give the C-4′ hydroxyethyl branched paulomycose moiety of PAU F (2). An acyltransferase Pau6 further decorates the C-4′ hydroxyethyl branch of paulomycose moiety of 2 by attaching various fatty acyl chains to 7′-OH to generate diverse PAUs. In addition, another acyltransferase Pau24 was proposed to be responsible for the 13-*O*-acetylation of PAUs.

Paulomycins (PAUs) are a group of glycosylated antibiotics with potential use to treat urethritis and *Chlamydia* infections, and are structurally defined by unique isothiocynate containing paulic acid and C-4′ hydroxyethyl branched paulomycose^1^,^2^,^3^,^4^ (Supplementary Fig. S1A). Several PAU analogs are distinguished from one another by the specific fatty acyl chains attached to the 7′-OH of their paulomycose moiety. C-4 branched octoses similar to paulomycose also occur in a variety of other antibiotics from gram positive bacteria, including antibacterial (avilamycins)^5^,^6^,^7^ and antitumor agents (quinocyclines,^8^,^9^ isoquinocyclines^10^,^11^ and trioxacarcins^12^,^13^) (Supplementary Fig. S1B). Installation of the C-4 acetyl branch has been proposed to be catalyzed by a putative protein complex (AviB1/AviB2) in avilamycin A biosynthesis using pyruvate as a donor^6^. However, relatively little is known about the following ketoreduction step converting the C-4 acetyl branch to the hydroxyethyl branch.

Besides antibiotics from gram positive bacteria, C-4 branched octoses have also been isolated in lipopolysaccharides of gram negative bacteria. C-4 hydroxyethyl branched (7*R*)-yersiniose A has been isolated from the *O*-antigens of several genera of gram negative bacteria including *Yersinia pseudotuberculosis* serovar VI^14^, *Yersinia frederiksenii*^15^, *Burkholderia brasiliensis*^16^, *Budvicia aquatica* 20186^17^ and *Pseudomonas mandelii*^18^. Its (7*S*)-isomer yersiniose B was observed in the *O*-antigen of *Yersinia enterocolitica*^19^ (Supplementary Fig. S1C). Unlike gram positive bacteria, a single TPP-dependent flavoprotein YerE is recruited to install the C-4 acetyl branch of yersiniose^20^. Unfortunately, the stereospecific ketoreduction step generating the two stereoisomers, yersiniose A (7*R*) and yersiniose B (7*S*), has yet not been characterized.

In the previous work^21^, we identified the PAU biosynthetic gene cluster from *Streptomyces paulus* NRRL 8115, which can produce PAU E (1), PAU F (2), PAU A (3), PAU B (4), paulomenol A (5) and paulomenol B (6) (Fig. 1). Among PAUs and paulomenols, compound 1 contains an octose with a C-4′ acetyl branch; while all the other compounds featuring the unique C-4′ hydroxyethyl branched paulomycose. The *S*-configuration of C-7′ was previously assigned by^1^H NMR of a paulomycose derivative hydrolyzed from 3 and 4 and the crystal structure of 5^22^. It was proposed that the AviB1/AviB2 homologs Pau11/Pau12 catalyze the attachment of the C-4′ acetyl branch to form the octose in compound 1. The C-4′ acetyl branch is then reduced to hydroxyethyl stereospecifically to afford the 7′-OH in 2, which will be decorated by various fatty acyl chains to generate diverse PAUs. In this work, we report Pau7 as a stereospecific ketoreductase converting the C-4′ acetyl branch of 1 to the C-4′ hydroxyethyl branch of 2, Pau6 as an acyltransferase loading different fatty acyl chains to 7′-OH and Pau24 as the enzyme catalyzing the acetylation of 13-OH.

## Results

### Pau7 catalyzes the 7′-ketoreduction of 1

**Figure 1:**
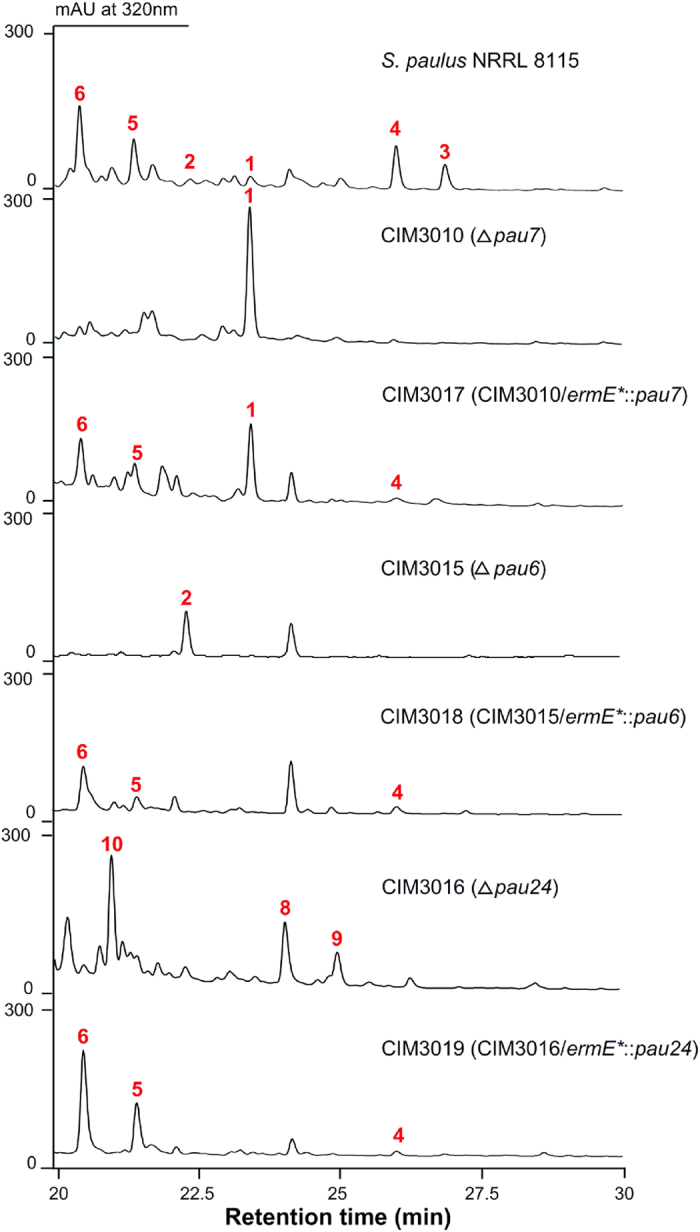
HPLC metabolic profiles of the *pau* gene inactivated mutants and the complemented strains. *S. paulus* NRRL 8115, the wild-type strain; CIM3010, the *pau7* inactivated mutant; CIM3017, complemented strain of CIM3010; CIM3015, the *pau6* inactivated mutant; CIM3018, complemented strain of CIM3015; CIM3016, the *pau24* inactivated mutant; CIM3019, complemented strain of CIM3016. 1, PAU E; 2, PAU F; 3, PAU A; 4, PAU B; 5, paulomenol A; 6, paulomenol B.

The gene *pau7* encodes a putative oxidoreductase belonging to the emerging aldo-keto reductase (AKR) superfamily^23^,^24^. Detailed analysis revealed that Pau7 possesses the conserved catalytic tetrad of the AKR proteins (Supplementary Fig. S4); and it represents a new subclass AKR5I since its protein sequence identity with the other enzymes from AKR5 family is higher than 40%, but less than 60%. The *pau7* inactivated mutant CIM3010 was constructed in the previous work^21^. HPLC analysis of CIM3010 revealed that it lost the capacity to produce all PAU analogs except 1 (Fig. 1), which was identified by its chemical formula C_29_H_36_N_2_O_16_S (determined by high-resolution electro spray ionization mass spectrometry, HR-ESI-MS, *m*/*z* 699.1716 for [M-H]^−^, calcd 699.1713) and its fragmentation pattern in tandem mass detection (Supplementary Fig. S5). Production of 4, 5 and 6 was partially restored by in trans complementation of *pau7* in CIM3010 (Supplementary Fig. S6), therefore excluding the possibility of a polar effect and confirming the involvement of Pau7 in PAU biosynthesis. Notably, paulomenols are converted from corresponding PAUs through hydrolysis of paulic acid at the late stages of fermentation.

**Figure 2:**
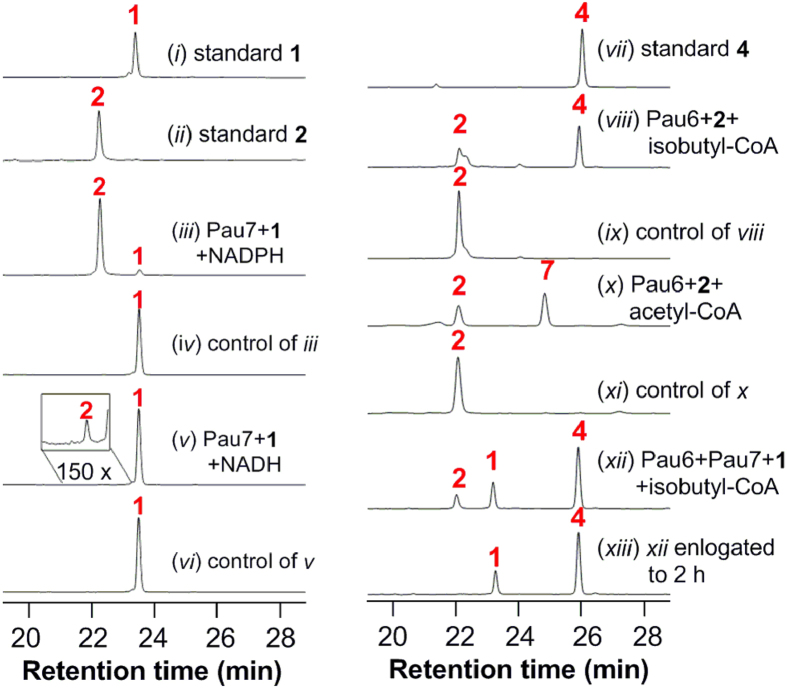
HPLC analysis of representative assays of Pau6 and Pau7. All control reactions were carried out with the corresponding boiled enzymes.

To verify Pau7 as the 7′-ketoreductase, we expressed this enzyme in *E. coli* as an N-His_6_-tagged protein and purified it by Ni-NTA affinity chromatography (Supplementary Fig. S7A). As expected, incubation of Pau7 with 1 and NADPH generated a new product with the same HPLC retention time (Fig. 2) and chemical formula as 2 (C_29_H_38_N_2_O_16_S, HR-ESI-MS, *m*/*z* 701.1859 for [M-H]^−^, calcd 701.1869) (Supplementary Fig. S6C). The dramatic loss of enzymatic activity, when NADH was used in place of NADPH (Fig. 2), indicates that Pau7 shows a distinct preference for NADPH as cofactor, as most of the other AKR enzymes^23^. The optimized reaction conditions of Pau7 were determined to be pH 7.5, 28 ^o^C. Steady-state kinetic analyses revealed Michaelis-Menten behavior for 1 with a *K*_m_ of 1.1 ± 0.2 mM and a *k*_cat_ of 19.5 ± 2.4 min^-1^ (Supplementary Fig. S7).

### Pau6 decorates the 7′-OH of paulomycose with different fatty acyl chains

After charactering Pau7 as the catalyst reducing the 7′-keto to 7′-OH, we sought to elucidate the mechanism of PAU structural diversification by addition of different fatty acyl chains to the 7′-OH. There are two putative acyltransferases encoded by the genes *pau6* and *pau24* within the *pau* cluster. Pau6 and Pau24 display high sequence identity to each other (39%) and to MppM (36.5% and 36.6%, respectively), the acyltransferase involved in mannopeptimycin biosynthesis^25^. To functionally distinguish the two enzymes, we constructed the *∆pau6* mutant CIM3015 and the *∆pau24* mutant CIM3016 by replacing *pau6* or *pau24* with an apramycin and a kanamycin resistance cassette, respectively (Supplementary Fig. S8). HPLC analysis of CIM3015 revealed that it produced none of the PAU analogs except 2 (Fig. 1), suggesting Pau6 as the 7′-OH acyltransferase. The identity of 2 was confirmed by HR-ESI-MS (*m*/*z* 701.1865 for [M-H]^−^, calcd 701.1869) and tandem mass analyses (Supplementary Fig. S9). Notably, production of 4, 5 and 6 could be restored by expressing *pau6* and *pau24* in CIM3015 and CIM3016 respectively in trans (Fig. 1), excluding the influence of polar effects.

To verify Pau6 as the 7′-OH acyltransferase, it was overexpressed as N-His_6_-tagged Pau6 in *S. lividans* TK24, purified by affinity chromatography (Supplementary Fig. S10A) and incubated with 2 and isobutyl-CoA. As anticipated, 2 was effectively converted to 4 (Fig. 2), which was confirmed by HR-ESI-MS (*m*/*z* 771.2281 for [M-H]^−^, calcd 771.2288) (Supplementary Fig. S10C). Pau6 was also incubated with 2 and acetyl-CoA, and a new compound possessing the same chemical formula as PAU D (7) (C_31_H_40_N_2_O_17_S, HR-ESI-MS, *m*/*z* 743.1981 for [M-H]^−^, calcd 743.1975, Supplementary Fig. S10E) was produced (Fig. 2), demonstrating the promiscuity of Pau6 towards different acyl-CoA substrates.

### Pau7 is a stereospecific ketoreductase

Conversion of 1 to 2 by Pau7 confirmed that it is a ketoreductase reducing the C-4′ acetyl branch to the hydroxyethyl branch of paulomycose. However, the catalytic stereospecificity of Pau7 is still a mystery since the configuration of C-7′ in compound 2 has not been determined. To interrogate the catalytic stereospecificity of Pau7, the coupled reaction of Pau6 and Pau7 was tested using 1 and isobutyl-CoA as substrates, in which 1 was converted to both 2 and 4 effectively in half an hour. When the reaction was elongated to two hours, 2 (generated by reduction of 1) was almost completely converted to 4 (Fig. 2), suggesting that 2 has the same 7′*S*-configuration as 4^22^, which indicates that Pau7 reduces the C-4′ acetyl branch stereospecfically to form the (7′*S*)-hydroxyethyl branch.

### Pau24 is responsible for the 13-OH acetylation

**Figure 3:**
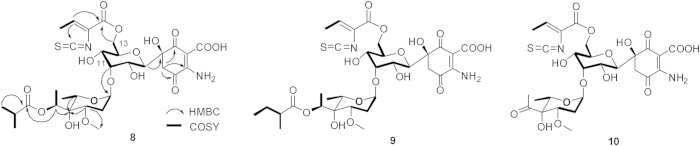
Chemical structures of 8, 9 and 10. The key COSY of 8, 9 and 10 are indicated by bold bonds; and the key HMBC correlations of 8 are marked with arrows.

Interestingly, the Δ*pau24* mutant CIM3016 abolished production of all PAU analogs but accumulated three new compounds (8, 9 and 10) (Fig. 1) possessing similar UV spectra (λ_max_ = 232 nm, 274 nm and 323 nm) as PAUs (Supplementary Figs S11B–S13B). HR-ESI-MS analysis of compound 8 resulted in an [M-H]^−^ ion at *m*/*z* 729.2191, consistent with the chemical formula C_31_H_42_N_2_O_16_S (calcd 729.2182) (Supplementary Fig. S11D) of deacetylated 4. Comparing ^1^H NMR data of 4 and 8 revealed that 8 lost the signals of 13-*O*-acetyl (Table S5), and the ^1^H NMR signals of 8 were almost identical to those of 4, except for the signals of the allose moiety^14^. The signals of H-9–H-12 of 8 clearly shift up-field, and in particular, the dramatic shift of H-11 from 4.81 ppm for 4 to 3.70 ppm for 8 indicates detachment of the pauloyl group. The coincident down-field shift of the H-13 signal (4.01 ppm for 4 to 4.16 ppm for 8) implies re-attachment of the pauloyl group by displacing the 13-*O*-acetyl group, which is confirmed by the HMBC correlation of 8 from H-13 (*δ*_*H*_: 4.16) to C-1″ (*δ*_*C*_: 161.2) (Supplementary Fig. S11K). Compound 8 was finally identified as 11-de-*O*-pauloyl-13-de-*O*-acetyl-13-*O*-pauloyl-PAU B based on its MS and NMR data (Fig. 3). The chemical formula of 9 was found to be C_32_H_44_N_2_O_16_S by HR-ESI-MS (*m*/*z* 743.2329 for [M-H]^−^, calcd 743.2339) (Supplementary Fig. S12D), which is consistent with deacetylated 3; similarly the chemical formula C_27_H_34_N_2_O_15_S of 10 (HR-ESI-MS, *m*/*z* 657.1599 for [M-H]^−^, calcd 657.1607) (Supplementary Fig. S13D) is the same as deacetylated 1. Compounds 9 and 10 were assigned as 11-de-*O*-pauloyl-13-de-*O*-acetyl-13-*O*-pauloyl-PAU A and 11-de-*O*-pauloyl-13-de-*O*-acetyl-13-*O*-pauloyl-PAU E analogous to 8 based on their tandem MS and NMR data (Fig. 3; Supplementary Figs S12 and S13). Finally, the antibiotic activities of the three new compounds 8, 9 and 10 were tested against several gram positive bacteria and no inhibition was observed (Table S8).

## Discussion

C-4 hydroxyethyl branched octoses distributed in both gram positive and gram negative bacteria. It was proposed that the C-4 hydroxyethyl branch was converted from C-4 acetyl branch by an uncharacterized ketoreduction step^20^. In this study, we characterized Pau7 as a stereospecific ketoreductase involved in paulomycin biosynthesis, which reduces the 7′-keto of 1 to form the C-4′ hydroxyethyl branched paulomycose moiety of 2. Bioinformatics analysis revealed that Pau7 belongs to AKR family and represents a new subgroup AKR5I. Surprisingly, no homolog of Pau7 was found in the biosynthetic gene clusters of avilamycin, kosinostatin (quinocycline B), trioxacarcin and yeriniose A, indicating that (i) the Pau7-like enzymes responsible for similar ketoreductions were encoded by separated genes or (ii) ketoreductases not belonging to AKR family were recruited in those cases.

Diverse PAUs were generated by decoration of the paulomycose 7′-OH with various fatty acyl chains^1^,^26^,^27^, indicating an acyltransferase with considerable substrate promiscuity has been recruited. Pau6 was characterized as the 7′-*O*-acyltransferase by both *in vivo* and *in vitro* evidence, and its flexibility towards different fatty acyl-CoA was demonstrated by the formation of 4 and 7 *in vitro*, when isobutyl-CoA or acetyl-CoA was used as the substrate.

The other acyltransferase, Pau24 was proposed to catalyze the acetylating at 13-OH based on the accumulation of 8, 9 and 10 in CIM3016. It is no doubt that the 13-*O*-acetylation step takes place prior to the formation of compound 1, but its exact timing cannot be determined at this stage. The 13-*O*-pauloyl moiety observed in compounds 8, 9 and 10 may be converted from their 11-*O*-pauloyl homologs by spontaneous transesterifications during fermentation (Supplementary Fig. S15), which has been similarly observed in many other cases^28^,^29^,^30^. Alternatively, the 13-*O*-pauloyl of 8, 9 and 10 may be formed by the pauloyl transferring enzyme, which adds pauloyl group to 11-OH of 13-*O*-acetylated substrates, but prefers 13-OH when 13-de-*O*-acetylated intermediates are accumulated in CIM3016. Since 13-*O*-pauloylated PAUs have never been isolated before, the antibacterial activities of 8, 9 and 10 were checked and resulted in no inhibition against the tested gram positive bacteria, indicating that 13-*O*-acetyl or the position of the pauloyl group is important to the bioactivity of PAUs.

**Figure 4:**
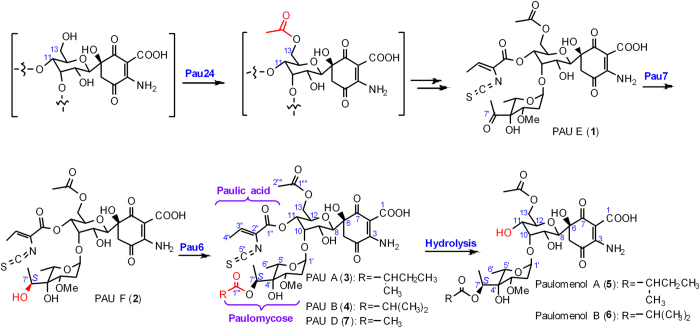
Proposed pathway for paulomycin biosynthesis. Pau24 is responsible for the acetylating at 13-OH; Pau7 catalyzes the stereospecific reduction of 7′-keto to (*S*)-7′-OH of the paulomycose; subsequently, Pau6 decorates 7′-OH with various fatty acyl chains to afford diverse PAUs.

Based on these results, we propose that Pau24 catalyzes the 13-*O*-acetylation step prior to the formation of 1. Compound 1 is then reduced stereospecifically to afford 2 by a novel AKR Pau7. Finally, Pau6 attaches different fatty acids to 7′-OH of 2’s paulomycose moiety to generate diverse PAU analogs (Fig. 4).

## Methods

### Bacterial strains and plasmids

Bacterial strains and plasmids used in this study are listed in Table S1.

### DNA manipulation and sequence analysis

General DNA manipulations were performed as described^31^. PCRs were performed with Taq DNA polymerase (TransGene, Beijing, China) or KOD-Plus DNA polymerase (Toyobo, Osaka, Japan) according to the manufacturers’ instructions. All PCR primers used in this study are listed in Table S2. Isolation of *Streptomyces* genomic DNA was performed according to the standard procedure^32^. Transformation of *Streptomyces* and *E. coli*-*Streptomyces* conjugations were carried out according to the standard protocols^32^. DNA sequencing was performed in Majorbio (Shanghai, China). BLASTP search was used to predict protein functions (http://blast.be-md.ncbi.nlm.nih.gov/Blast.cgi). Multiple alignments were performed with CLUSTALW.

### Construction of the ∆*Pau6* mutant CIM3015

The *pau6* gene inactivated mutant was constructed by replacing the target gene with the apramycin resistant gene cassette (*aac(3)IV*) *via* double-crossover recombination (Supplementary Fig. S9). To construct the *pau6* mutant, the two fragments flanking *pau6* were amplified by PCR using primer pair *pau4*-S and *pau6*-R for the 1.7-kb upstream fragment and primer pair *pau6*-S and *pau8*-S for the 1.9-kb downstream fragment. The two fragments were then inserted into the *Bln*I and *Mun*I sites of pCIM2004 respectively *via* LIC strategy^33^ to generate pCIM3019. Introduction of plasmid pCIM3019 into *S. paulus* NRRL 8115 was carried out by *E. coli-Streptomyces* conjugation. Exconjugants with apramycin resistance and without blue pigment were selected as the desired ∆*Pau6* mutants. Genotype confirmation of the ∆*Pau6* mutants were carried out by PCR with primers *pau6-*ES and *pau6-*ER, and one of the confirmed mutant was termed as *S. paulus* CIM3015. (Supplementary Fig. S8).

### Construction of the ∆*Pau24* mutant CIM3016

The *pau24* gene disruption mutant was constructed by replacing the target gene with the kanamycin resistant gene cassette (*aph*) (Supplementary Fig. S8). The 1.2-kb upstream framgent of *pau24* was amplified using primer pair *pau23*-ES and *pau23*-ER. The 1.3-kb downsteam fragment of *pau24* was amplified using primer pair *pau24*D-S and *pau25*-ER. The two fragments were inserted into *Pst*I*/Bam*HI and *Kpn*I/*Eco*RI sites of pUC119::KanR respectively to generate pCIM3020. The 3.5-kb mutant allele containing the up- and down-stream fragments of *pau24* and the kanamycin resistance cassette was excised by *Pst*I/*Eco*RI and inserted into the same sites of pKC1132 to afford pCIM3021. Plasmid pCIM3021 was then introduced into *S. paulus* NRRL 8115 *via E. coli-Streptomyces* conjugation. Exconjugants with kanamycin resistance and apramycin sensitivity were selected as the desired ∆*Pau24* mutants. Genotype confirmation of the ∆*Pau24* mutants were carried out by PCR using primers *pau23*-ES and *pau25*-ER and subsequent *Bam*HI digestion, and one of the confirmed mutant was designated as *S. paulus* CIM3016 (Supplementary Fig. S8).

### Complementation of CIM3010, CIM3015 and CIM3016

To complement the Δ*pau7* mutant CIM3010^21^, the 1.0-kb DNA fragment containing the whole *pau7* gene was amplified from the *S. paulus* NRRL 8115 genome using primer pair *pau7-*ES/*pau7*-ER and inserted into the *Eco*RV site of pBluscript II SK(+). After verifying the DNA fidelity by sequencing, the 1.0-kb *pau7* fragment was excised by *Nde*I/*Bam*HI and inserted into the same sites of pUWL201PW-oriT to afford pCIM3022. Introduction of pCIM3022 into CIM3010 by *E. coli-Streptomyces* conjugation generated the *pau7* complemented strain CIM3017.

Similary, the 1.3-kb *pau6* fragment, was PCR cloned using primer pair *pau6*-ES/*pau6*-ER, inserted into pBluscript II SK(+) for sequencing, excised respectively by *Nde*I/*Eco*RI, and inserted into the same sites of pUWL201PW-oriT to generate pCIM3023. The Δ*pau6* complemented strain CIM3018 was obtained by introducing pCIM3023 into CIM3015.

To complement the Δ*pau24* mutant CIM3016, the 1.3-kb DNA fragment containing the whole *pau24* gene was amplified using primer pair *pau24-*ES/*pau24*-ER and inserted into the *Eco*RV site of pBluscript II SK(+). After verifying the DNA fidelity, the 1.3-kb *pau24* fragment was excised by *Nde*I/*Eco*RI and inserted into the same sites of pSET152-ermE* to afford pCIM3024. Transformation of pCIM3024 into CIM3016 generated the Δ*pau24* complemented strain CIM3019.

### Expression and purification of Pau7

The 1.0-kb *pau7* gene amplified with primer pair *pau7*-ES/*pau7*-ER was cloned into pBluscript II SK(+) and sequenced. The sequence correct fragment was then excised by *Nde*I/*Bam*HI and inserted into the same sites of pET-28a to afford pCIM3025. A single transformant of *E. coli* BL21 (DE3)/pCIM3025 was inoculated into LB (with 100 *μ*g/mL kanamycin) and cultured overnight at 37 ^o^C, 220 rpm. The overnight culture was used to inoculate LB medium (with 100 *μ*g/mL kanamycin) at 1:100 dilution and incubated at 28 ^o^C, 220 rpm until OD_600_ reached 0.6. Expression of Pau7 was then induced by the addition of isopropyl-*β*-thiogalactoside (IPTG) at a final concentration of 0.1 mM and cultured at 18 ^o^C, 180 rpm for further 18–20 hours.

Pau7 was purified using the Ni-NTA affinity column following the instruction of the manufacture (Novagen). All steps were conducted under 4 ^o^C. Briefly, the cells were harvested by centrifugation and were then resuspended in lysis buffer (20 mM Tris-HCl, 500 mM NaCl, 5 mM imidazole, and 5% glycerol, pH 7.9). After ultrasonication, cell debris was removed by centrifugation (16,000 × g, 30 min). The supernatant containing His-tagged proteins was loaded onto the Ni-NTA affinity column, washed with washing buffer (lysis buffer with 60 mM imidazole), and elution buffer (lysis buffer with 250 mM imidazole) stepwise. The purified protein was desalted and concentrated by ultracentrifugation and stored at −80 ^o^C in 100 mM HEPES buffer (pH 7.5) with 500 mM NaCl and 20% glycerol. All protein concentrations in this study were measured using Bradford method.

### Expression and purification of Pau6

The genes *pau6* was amplified from *S. paulus* NRRL8115 genome using primer pair *pau6*-ES/*pau6*-ER and cloned into pBluscript II SK(+) for sequencing. The sequence correct fragment was then excised by *Nde*I/*Eco*RI and inserted into the same sites of pPWW50-Gen Poly to afford pCIM3026. The DNA fragment containing *oriT* which was amplified by primer pair *oriT*-F/*oriT*-R and inserted into the *Kpn*I site of pCIM3026 *via* LIC strategy to generate pCIM3027. Plasmid pCIM3027 was introduced into *S. lividans* TK24 to generate the desired *Pau6* heterologous expression strain CIM3020. CIM3020 was incubated in YEME medium (with 12.5 *μ*g/mL thiostrepton) for 3 days and the cells were harvested by centrifugation and then resuspended in lysis buffer. Pau6 was purified with the Ni-NTA affinity column following the same procedure as that for Pau7 purification and stored at −80 ^o^C in 100 mM Tris-HCl buffer (pH 7.5) with 20% glycerol until use.

### Enzymatic assays of Pau7

The assays of Pau7 were performed in a 50 *μ*l mixture containing 100 mM HEPES buffer (pH 7.5), 500 mM NaCl, 2 mM NADPH, 0.2 mM 1, and 6 *μ*M Pau7 at 28 ^o^C for 30 min. The reaction was quenched by three volumes of acetone. Temperature optimization of the Pau7 *in vitro* assay was performed in a 25 *μ*L reaction mixture containing 500 mM NaCl, 2 mM NADPH, 80 *μ*M 1 and 2 *μ*M Pau7 in 100 mM HEPES buffer (pH 7.0) for 30 min. Optimizing pH values of the Pau7 were carried out at 28 °C in buffers of pH ranging from 6.0 to 8.0 (50 mM phosphate buffer for pH 6.0–7.0, and 100 mM HEPES buffer for pH 7.0–8.0) for 30 min; each 25 *μ*L reaction mixture contained 500 mM NaCl, 2 mM NADPH, 40 *μ*M 1 and 2 *μ*M Pau7. Determination of kinetic parameters for 1 was carried out with saturating NADPH (4 mM). Compound 1 was set as a variable substrate in concentrations of 0.02, 0.04, 0.09, 0.18, 0.36, 0.72, 1.4, and 2.8 mM. Enzyme assay was performed in HEPES buffer (100 mM, pH 7.5) containing 500 mM NaCl and 2 *μ*M Pau7 at 28 ^o^C for 2.5 min in triplicate.

### Enzymatic assays of Pau6

The assays of Pau6 were performed in a 50 *μ*l mixture containing 100 mM Tris-HCl buffer (pH 7.5), 100 mM MgCl_2_, 10 mM dithiothreitol, 500 mM NaCl, 2mM isobutyl-CoA or acetyl-CoA, 80 *μ*M 2, and 2 *μ*M Pau6 at 28 ^o^C for 30 min. The Pau7 and Pau6 coupled assays were carried out as follows: The general reaction of Pau7 was quenched and extracted by 3 volume of ethyl acetate, dried in *vacuo*, then re-dissolved used as the substrates for Pau6 *in vitro* assays.

### Production of paulomycins

For paulomycins production, 50 *μ*L spores of *S. paulus* NRRL 8115 and the mutant strains were inoculated into GS-7 medium^34^ and cultured at 28 °C for 2 days. The resulting seed culture was inoculated into 50 mL medium R5α^35^ at 2% ratio (v/v) and cultured for 4 days. The fermentation broth was harvested by centrifugation and extracted with 50 mL ethyl acetate for three times. After dried in *vacuo*, it was re-dissolved in 1 mL acetonitrile and subjected to HPLC analysis.

### Isolation of Compounds 8, 9 and 10

The supernatant from 30 L R5α culture broth of CIM3016 was collected and extracted with 30 L ethyl acetate for three times. After concentrated in *vacuo*, it was dissolved in a small amount of ethyl acetate and developed on silica gel column chromatography, eluted stepwise with 500 mL petroleum ether, 1000 mL ethyl acetate, 1000 mL 50% ethyl acetate/methanol, and 500 mL methanol. The ethyl acetate fraction containing compounds 8, 9 and 10 was concentrated in *vacuo.* After dissolving in a small volume of acetonitrile, compounds 8, 9 and 10 were further purified by semi-preparative HPLC (Zorbax SB-C18, 5 *μ*m, 9.4 × 250 mm, Agilent, Santa Clara, CA, USA) eluted with acetonitrile:water (40:60) at a flow rate of 2 mL/min. Compounds 8, 9 and 10 were eluted at 9.2 min, 11.1 min and 6.3 min, respectively. Finally, 3.0 mg of 8, 2.0 mg of 9 and 1.2 mg of 10 were obtained.

### Isolation of Compounds 4 and 6

Compounds 4 and 6 were isolated from 30 L culture broth of *S. paulus* NRRL8115 in a similar manner as compounds 8, 9 and 10. The supernatant was extracted with 30 L ethyl acetate for three times. After concentrated in vacuo and dissolved in a small amount of ethyl acetate, it was loaded on silica gel column chromatography and developed with 500 mL petroleum ether, 1000 mL ethyl acetate, 1000 mL 50% ethyl acetate/methanol, and 500 mL methanol. The ethyl acetate fraction containing compounds 4 and 6 was then concentrated and further purified by semi-preparative HPLC (Zorbax SB-C18, 5μm, 9.4 × 250 mm, Agilent, Santa Clara, CA, USA) using the same program as that for compounds 8, 9 and 10. Compounds 4 and 6 were eluted at 7.8 min and 6.7 min, respectively. At last, 3.0 mg of 4 and 4.0 mg of 6 were obtained.

### Antibacterial assays

Antibacterial activity was determined by agar dilution method according to the method as described^2^,^36^. *Staphylococcus pneumonia, Staphylococcus pyogenes, Staphylococcus epidermidis, Staphylococcus aureus and Bacillus subtilis* were used as the tested bacteria. After incubation for 24 h at 37 °C, the MICs were calculated (Table S3).

### Spectroscopic analysis

HPLC analyses were carried out with an Apollo C18 column (5 *μ*m, 4.6 mm × 250 mm, Alltech, Deerfield, IL, USA) on a Shimadzu HPLC system (Shimadzu, Kyoto, Japan). Briefly, the column was developed with solution A (water with 0.1% trifluoroacetic acid) and acetonitrile at a flow rate of 0.8 mL/min. The percentage of acetonitrile was changed using the following gradient: 0–5 min, 10%; 5–25 min, 10%–90%; 25–30 min, 90%–100%; 30–35 min, 100%. The detection wavelength was 320 nm. LC-MS analyses were performed on an Agilent 1260/6460 Triple-Quadrupole LC/MS system (Santa Clara, CA, USA) with an electrospray ionization source. HR-ESI-MS was performed on an Agilent 1260 HPLC/6520 QTOF-MS instrument (Santa Clara, CA, USA). NMR spectra were recorded at room temperature on a Bruker-500 NMR spectrometer (Billerica, MA, USA).

## Additional Information

**How to cite this article**: Li, J. *et al.* Involvement of an octose ketoreductase and two acyltransferases in the biosynthesis of paulomycins. *Sci. Rep.* **6**, 21180; doi: 10.1038/srep21180 (2016).

## Supplementary Material

Supplementary Information
