# Supplementary material for: Involvement of an octose ketoreductase and two acyltransferases in the biosynthesis of paulomycins
Source: Sci Rep. 2016 Feb 15;6:21180. doi: 10.1038/srep21180 (PMC4753412; doi:10.1038/srep21180)
Supplement: Supplementary Information [file srep21180-s1.pdf]

Supplementary Information

Involvement of an octose ketoreductase and two acyltransferases in the biosynthesis of paulomycins

Jine Li,[†,‡] Min Wang,[†,‡] Yong Ding,[†,§] Tang Yue[#], Zhiguo Zhang*[,#] and Yihua Chen*[,†]

[†]State Key Laboratory of Microbial Resources, Institute of Microbiology, Chinese Academy of Sciences, Beijing 100101, China

[§]University of Chinese Academy of Sciences, Beijing, 110039, China

[#]QiLu University of Technology, Jinan, Shandong, 250353,China

Corresponding Author

[*]E-mail: chenyihua@im.ac.cn;

zzg@qlu.edu.cn

Author Contributions

[‡]These authors contributed equally.

TABLE S1. Bacterial strains and plasmids.

Strains or plasmids	Characteristics	Reference or source
Escherichia coli		
JM109	General cloning host	Invitrogen
ET12567	Strain deficient in DNA methylation	[1]
ET12567/pUZ8002	Donor strain for *E. coli-Streptomyces* conjugation	[1]
BL21 (DE3)	Host strain for protein expression	Novagen
Streptomyces		
S. paulus		
NRRL8115	*S. paulus* wild-type	NRRL
CIM3010	*S. paulus* NRRL8115 Δ*pau7::aac(IV)3*	[2]
CIM3015	*S. paulus* NRRL8115 Δ*pau6::aac(IV)3*	this study
CIM3016	*S. paulus* NRRL8115 Δ*pau24::aac(IV)3*	this study
CIM3017	*S. paulus* CIM3010 harboring pCIM3022	this study
CIM3018	*S. paulus* CIM3015 harboring pCIM3023	this study
CIM3019	*S. paulus* CIM3016 harboring pCIM3024	this study
lividans		
TK24	*S. lividans* wild-type	[1]
CIM3020	*S. lividans* TK24/pCIM3027	this study
CIM3021	*S. lividans* TK24/pCIM3029	this study
Staphylococcus		
S. pneumoniae		[3]
S. pyogenes		[3]
S. epidermidis		ATCC 35984
S. aureus		[3]
Bacillus subtilis		[3]
**Plasmids**		
pET28a	Kan^r, protein expression vector	Novagen
pBluescript II KS(+)	Amp^r, replicating vector in *E. coli*	Stratagene
pMD19-T	Amp^r, T-vector for DNA cloning in *E. coli*	Takara
pUC119::KanR	Amp^r, Kan^r, replicating vector in *E. coli*	[4]
pSET152::ermE*	*E.coli-Streptomyces* shuttle vector, with *ermE** promoter	[5]
pKC1132	Apr^r , *E.coli-Streptomyces* shuttle vector	[1]

pUWL201PW-oriT	Amp^r/Tsr^r, replicating vector in *Streptomyces*	[6]
pPWW50-Gen Poly	Amp^r/Tsr^r, replicating vector in *Streptomyces*	[7]
pCIM2004	Apr^r, *ermEp*-idgS-sfp, E.coli-Streptomyces* shuttle vector, nonreplicative in *Streptomyces*	[8]
pCIM3019	pCIM2004 with *pau6*:: *aac(IV)3*	this study
pCIM3020	pUC119::KanR with *pau24*:: *neo*	this study
pCIM3021	pKC1132 with *pau24*:: *neo*	this study
pCIM3022	pUWL201PW-oriT with *pau7* under the control of *ermE**	this study
pCIM3023	pUWL201PW-oriT with *pau6* under the control of *ermE**	this study
pCIM3024	pSET152::ermE* with *pau24* under the control of *ermE**	this study
pCIM3025	pET28a derived, for expression of the *pau7* gene	this study
pCIM3026	pPWW50-Gen Poly with *pau6* under the control of *ermE**	this study
pCIM3027	pCIM3026 with *oriT*	this study

TABLE S2. Primers used in this study.

Primers	Sequence (5'-3')	Restriction sites
***pau4*-S**	CTTCCATGGGCACGCCCTAGGAGCACCTTTCACGGGTACATCAG	*Bln*I
***pau6*-R**	ATCCCTTAACGTGAGCCTAGGGGCGACCGTGGCCTTTGTAC	*Bln*I
***pau6*-S**	CAGTCGATTGGCTGACAATTGGGTGCGGCTGGGAGACATCA	*Mun*I
***pau8*-S**	CTTGCTAGCAGATGTCAATTGGATCCGCTCATCGTTCGTCAA	*Mun*I
***pau6*-ES**	AAAACTGCAGCATATGGCCTCGGACGCAACTTC	*Nde*I
***pau6*-ER**	CGGAATTCGTCGGCTCCTGACGGACGAAG	*Eco*RI
***pau7*-ES**	AAAACTGCAGCATATGAACAGGATTCCCTCCGTC	*Nde*I
***pau7*-ER**	CGGGATCCTCCTGGTGCCGAACGTGAAG	*Bam*HI
***pau23*-ES**	AAAACTGCAGCATATGCGTGGAATTCTCCTGGC	*Pst*I
***pau23*-ER**	CGGGATCCATTCAATAGTGGACGCACCG	*Bam*HI
***pau24*D-S**	GGGGTACCACACCTCCGCCCCTGTATGAC	*Kpn*I
***pau25*-ER**	GGAATTCAGGTCCTCCATCGCCTCGTC	*Eco*RI
***oriT*-F**	TGCTCGGGTCGGGCTGGGGTACCTGGTTGGCTTGGTTTCATC	*Kpn*I
oriT-R	GCACTTCGTGCAGGCGGTACCTGCCAAAGGGTTCGTGTAG	*Kpn*I

Notes: The designed restriction sites in the primers for gene cloning were underlined.

TABLE S3. ^{1}H NMR (500 MHz) and ^{1}H-^{1}H COSY data of compound **4** in acetone-d_6.

Position	δ_H (ppm)	^{1}H-^{1}H COSY
1		
2		
3		
4		
5	3.20 (2H, m)	
6		
7		
8	3.85 (1H, d, J = 10 Hz)	H-9
9	3.72 (1H, dd, J = 10 Hz, 2Hz)	H-8, H-10
10	4.31 (1H, m)	H-9, H-11
11	4.81 (1H, dd, J = 10 Hz, 2Hz)	H-10, H-12
12	4.19 (1H, m)	H-11, H-13
13	4.01 (2H, m)	H-12
1′	4.95 (1H, m)	H-2′
2′	1.90, 2.23 (2H, m)	H-1′, H-3′
3′	3.66 (1H, dd, J = 4.8Hz, 11.0Hz)	H-2′
3′-OMe	3.33 (3H, s)	
4′		
5′	4.52 (1H, q, J = 6.4 Hz)	H-6′
6′	1.24 (3H, d, J = 6.4 Hz)	H-5′
7′	5.35 (1H, q, J = 6.8 Hz)	H-8′
8′	1.28 (3H, d, J = 6.8 Hz)	H-7′
1″		
2″		
3″	6.83 (1H, q, J = 7 Hz)	H-4″
4″	1.95 (3H, d, J = 7 Hz)	H-3″
5″		
1‴		
2‴	2.65 (1H, m)	H-3‴, H-4‴
3‴	1.21 (3H, d, J = 7 Hz)	H-2‴, H-4‴
4‴	1.19 (3H, d, J = 7 Hz)	H-2‴, H-3‴
5‴		
1⁗		
2⁗	1.99 (3H, s)	

TABLE S4. ^{1}H NMR (500 MHz), ^{13}C NMR (125 MHz), ^{1}H-^{1}H COSY and HMBC data of compound **6** in acetone-d_6.

Position	δ_H (ppm)	δ_C (ppm)	^{1}H-^{1}H COSY	HMBC
1		170.1		
2		100.7		
3		160.2		
4		188.8		
5	3.15 (2H, m)	47.9		C-4, C-7, C-3, C-6
6		78.1		
7		198.1		
8	3.68 (1H, d, J = 10 Hz)	78.1	H-9	C-7, C-6
9	3. 48 (1H, m)	69.7	H-8, H-10	C-8
10	4.08 (1H, m)	79.8	H-9, H-11	C-9, C-11
11	3.44 (1H, m)	67.8	H-10, H-12	C-12
12	3.80 (1H, m)	74.6	H-11, H-13	C-11
13	4.05 (2H, m)	63.8	H-12	C-1′′′′, C-12
1′	5.15 (1H, m)	99.5	H-2′	C-3′
2′	1.92, 2.27 (2H, m)	30.3	H-1′, H-3′	C-1′,C-3′
3′	3.66 (1H, dd, J = 4.8Hz, 11.0Hz)	74.6	H-2′	C-3′-OMe
3′-OMe	3.32 (3H, s)	56.7		C-4′
4′		73.8		
5′	4.52 (1H, q, J = 6.3 Hz)	67.8	H-6′	C-6′
6′	1.27 (3H, d, J = 6.3 Hz)	15.4	H-5′	C-5′
7′	5.35 (1H, q, J = 6.8 Hz)	70.1	H-8′	C-8′, C-4′, C-1′′′, C-5′
8′	1.29 (3H, d, J = 6.8 Hz)	15.6	H-7′	C-7′
1′′′		175.8		
2′′′	2.63 (1H, m)	34.4	H-3′′′, H-4′′′	C-1′′′, C-3′′′, C-4′′′
3′′′	1.22 (3H, d, J = 7.0 Hz)	19.1	H-4′′′	C-2′′′, C-4′′′
4′′′	1.97 (3H, d, J = 7.0 Hz)	18.9	H-3′′′	C-2′′′, C-3′′′
5′′′				
1′′′′		171.2		
2′′′′	2.04 (3H, s)	20.2		C-1′′′′

TABLE S5. ^{1}H NMR (500 MHz), ^{13}C NMR (125 MHz), ^{1}H-^{1}H COSY and HMBC data of compound **8** in acetone-d_6.

Position	δ_H (ppm)	δ_C (ppm)	^{1}H-^{1}H COSY	HMBC
1		153.9		
2		123.3		
3		159.2		
4		188.5		
5	3.11, 3.31 (2H, s)	48.6		C-4, C-7, C-3, C-6
6		77.9		
7		198.0		
8	3.73 (1H, d, *J* = 10 Hz)	78.0	H-9	C-6, C-7
9	3.56 (1H, m)	69.3	H-8, H-10	C-8
10	4.10 (1H, m)	79.1	H-9, H-11	C-1′, C-8, C-9, C-11
11	3.70 (1H, m)	66.8	H-10, H-12	C-12
12	3.91 (1H, m)	74.3	H-11, H-13	C-11
13	4.16 (2H, m)	64.5	H-12	C-1″
1′	5.18 (1H, m)	99.0	H-2′	C-3′
2′	1.90, 2.22 (2H, m)	30.0	H-1′, H-3′	C-1′, C-3′
3′	3.64 (1H, dd, *J* = 11 Hz, 5Hz)	74.3	H-2′	C-3′-OMe
3′-OMe	3.29 (3H, s)	56.3		C-3′
4′		73.4		
5′	4.52 (1H, q, *J* = 6.4 Hz)	67.2	H-6′	C-1′, C-4′, C-6′
6′	1.26 (3H, d, *J* = 6.4 Hz)	15.0	H-5′	C-5′, C-4′
7′	5.36 (1H, q, *J* = 6.8 Hz)	69.8	H-8′	C-8′, C-5′, C-4′, C-1‴
8′	1.28 (3H, d, *J* = 6.8 Hz)	15.2	H-7′	C-7′, C-4′
1″		161.2		
2″		123.3		
3″	6.73 1H, (q, *J* = 7 Hz)	135.7	H-3″	C-4″, C-2″, C-1″
4″	1.97 (3H, d, *J* = 7 Hz)	13.9	H-4″	C-2″, C-3″
5″		142.1		
1‴		175.8		
2‴	2.64 (1H, m)	34.0	H-3‴, H-4‴	C-1‴, C-3‴, C-4‴
3‴	1.20 (2H, d, *J* = 7 Hz)	18.7	H-2‴, H-4‴	C-1‴, C-2‴, C-4‴
4‴	1.18 (3H, d, *J* = 7 Hz)	18.5	H-2‴, H-3‴	C-1‴, C-2‴, C-3‴

TABLE S6. ^{1}H NMR (500 MHz) and ^{1}H-^{1}H COSY data of compound **9** in acetone-d_6.

Position	δ_H (ppm)	^{1}H-^{1}H COSY
1		
2		
3		
4		
5	3.11, 3.29 (2H, s)	
6		
7		
8	3.73 (1H, d, J = 10 Hz)	H-9
9	3.56 (1H, m)	H-8, H-10
10	4.11 (1H, m)	H-9, H-11
11	3.70 (1H, m)	H-10, H-12
12	3.90 (1H, m)	H-11, H-13
13	4.17 (2H, m)	H-12
1′	5.18 (1H, dd, J = 4 Hz, 2 Hz)	H-2′
2′	1.93, 2.22 (2H, m)	H-1′, H-3′
3′	3.65 (1H, dd, J = 11 Hz, 5Hz)	H-2′
3′-OMe	3.33 (3H, s)	
4′		
5′	4.52 (1H, q, J = 6.5 Hz)	H-6′
6′	1.26 (3H, d, J = 6.5 Hz)	H-5′
7′	5.36 (1H, q, J = 6.8 Hz)	H-8′
8′	1.29 (3H, d, J = 6.8 Hz)	H-7′
1″		
2″		
3″	6.73 (1H, q, J = 7 Hz)	H-3″
4″	1.97 (3H, d, J = 7 Hz)	H-4″
5″		
1‴		
2‴	2.45 (1H, m)	H-5‴
3‴	1.53, 1.72 (2H, m)	H-4‴
4‴	0.97 (3H, t, J = 7 Hz)	H-3‴
5‴	1.18 (3H, d, J = 7 Hz)	H-2‴, H-4‴

TABLE S7. ^{1}H NMR (500 MHz) and ^{1}H-^{1}H COSY data of compound **10** in acetone-d_6.

Position	δ_H (ppm)	^{1}H-^{1}H COSY
1		
2		
3		
4		
5	3.56 (2H, m)	
6		
7		
8	3.85 (1H, d, J = 10 Hz)	H-9
9	3.6 (1H, m)	H-8, H-10
10	4.13 (1H, m)	H-9, H-11
11	3.74 (1H, dd, J = 10 Hz, 2Hz)	H-10, H-12
12	4.0 (1H, m)	H-11, H-13
13	4.19 (2H, m)	H-12
1′	5.29 (1H, d, J = 3.3 Hz)	H-2′
2′	1.80, 2.30 (2H, m)	H-1′, H-3′
3′	4.18 (1H, m)	H-2′
3′-OMe	3.23 (3H, s)	
4′		
5′	4.60 (1H, q, J = 6.4 Hz)	H-6′
6′	0.97 (3H, d, J = 6.4 Hz)	H-5′
7′		
8′	2.27 (3H, s)	
1″		
2″		
3″	6.73 (1H, q, J = 7 Hz)	H-4″
4″	1.96 (3H, d, J = 7 Hz)	H-3″
5″		

TABLE S8. Antibacterial activities of PAUs.

Organisms	MIC[a] (μg/ml)				
	**3**	**4**	**9**	**8**	**10**
S. pneumoniae	0.05	0.05	>100	>100	>100
S. pyogenes	0.025	0.025	>50	>50	>100
S. epidermidis	0.05	0.05	>100	>100	>100
S. aureus	0.1	0.1	>100	>100	>100
Bacillus subtilis	0.1	0.1	>100	>100	>100

[a] MIC: Minimum inhibitory concentration (μg/ml) determined by agar dilution method as described[9].

Reference

1. Kieser, T., Bibb, M. J., Buttner, M. J., Chater, K. F. & Hopwood, D. A. in *Practical Streptomyces genetics* (The John Innes Foundation: Norwich, 2000).

2. Li, J., Xie, Z., Wang, M., Ai, G. & Chen, Y. Identification and analysis of the paulomycin biosynthetic gene cluster and titer improvement of the paulomycins in *Streptomyces paulus* NRRL 8115. *PloS One*, **10**, e0120542 (2015).

3. Lu, C., Liao, G., Zhang, J & Tan, H. Identification of novel tylosin analogues generated by a wblA disruption mutant of *Streptomyces ansochromogenes*. *Microb. Cell Fact.* **14**, 173 (2015).

4. Liu, G., Tian, Y., Yang, H. & and Tan, H. A pathway-specific transcriptional regulatory gene for nikkomycin biosynthesis in *Streptomyces ansochromogenes* that also influences colony development. *Mol. Microbiol*. **55**, 1855-1866 (2005).

5. Smanski, M. J. *et al.* Dedicated ent-kaurene and ent-atiserene synthases for platensimycin and platencin biosynthesis. *Proc. Natl. Acad. Sci. USA.* **108**, 13498-13503 (2011).

6. Doumith, M. *et al.* Analysis of genes involved in 6-deoxyhexose biosynthesis and transfer in *Saccharopolyspora erythraea*. *Mol.Gen.Genet*. **264**, 477-485 (2000).

7. Zhang, C., Fu, Q., Albermann, C., Li, L. & Thorson, J. S. The *in vitro* characterization of the erythronolide mycarosyltransferase EryBV and its utility in macrolide diversification. *Chembiochem*. **8**, 385-390 (2007).

8. Li, P. *et al.* An efficient blue-white screening based gene inactivation system for *Streptomyces*. *Appl. Microbiol. Biotechnol.* **99**, 1923-1933 (2015).

9. Argoudelis, A. D., Baczynskyj, L., Mizsak, S. A., Shilliday, F. B. & Wiley, P. F. Structural relationships between senfolomycins and paulomycins. *J. Antibiot.* **41**, 1212-1222 (1988).

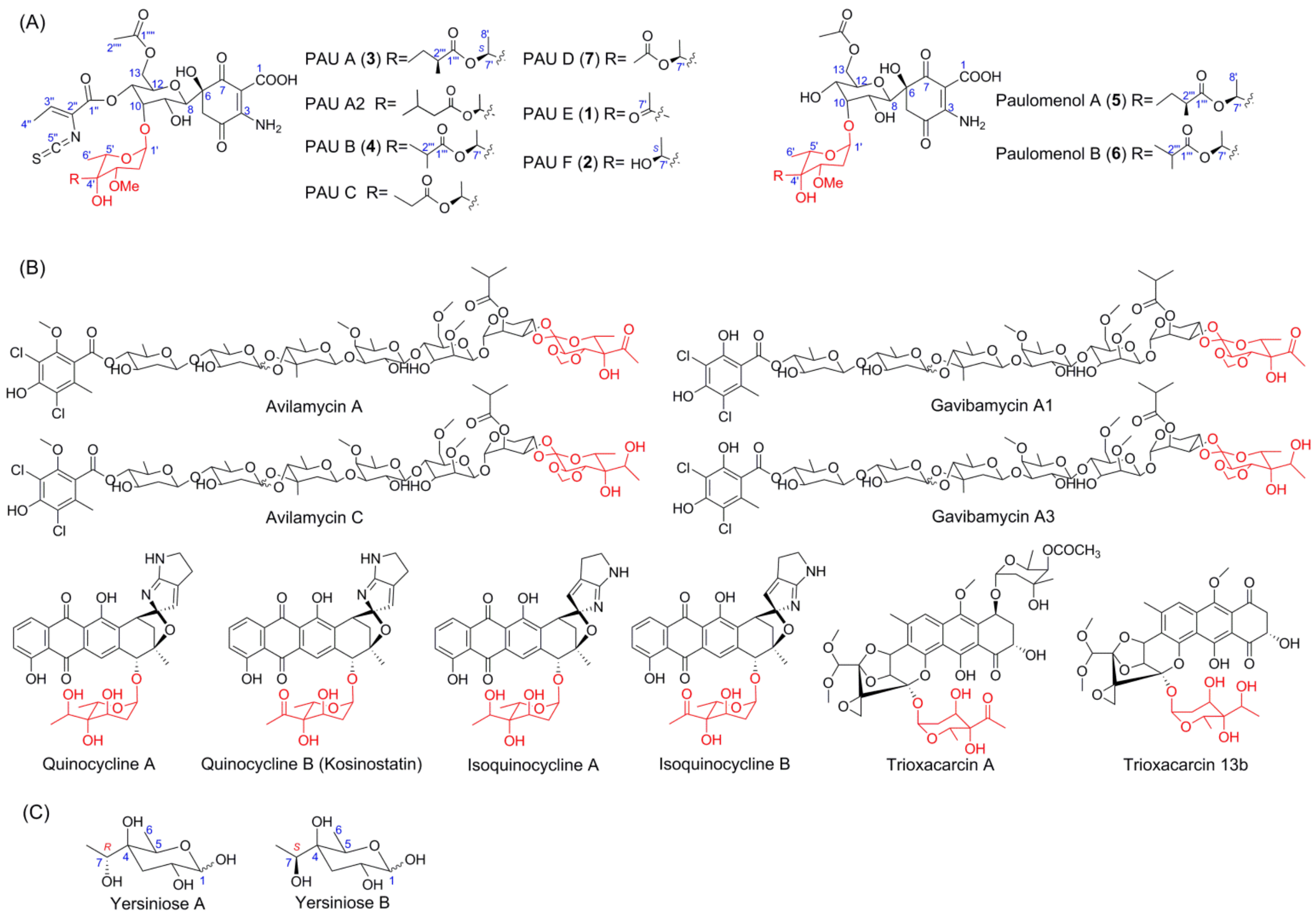

Figure S1. Structures of natural products with C4-branched octoses. PAU, paulomycin.

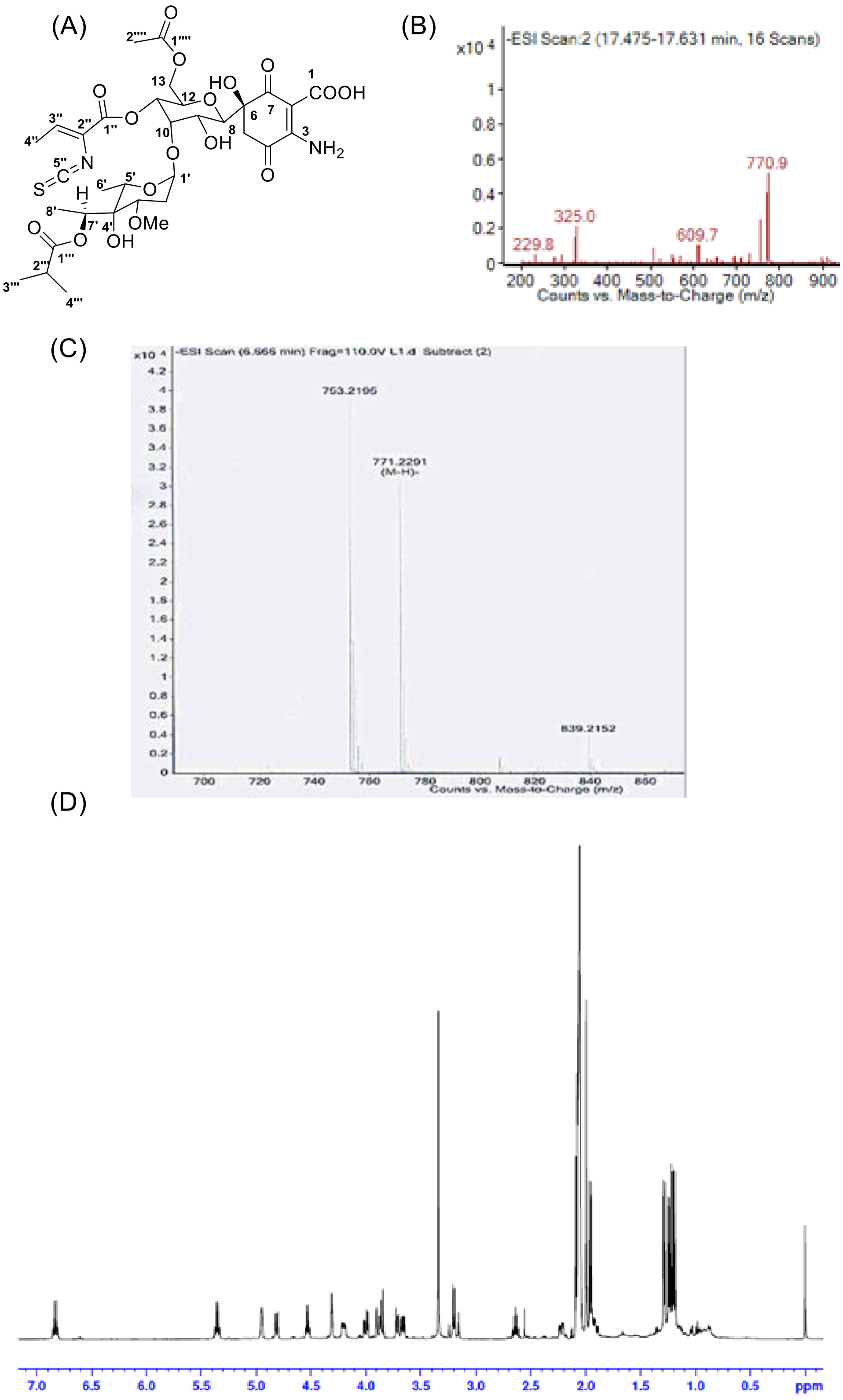

Figure S2-1. Spectral data of compound **4**. (A) Structure of compound **4**. (B) ESI-MS spectrum of **4**. (C) HR-ESI-MS spectrum of **4**. (D) ^{1}H NMR spectrum of **4**.

(E)

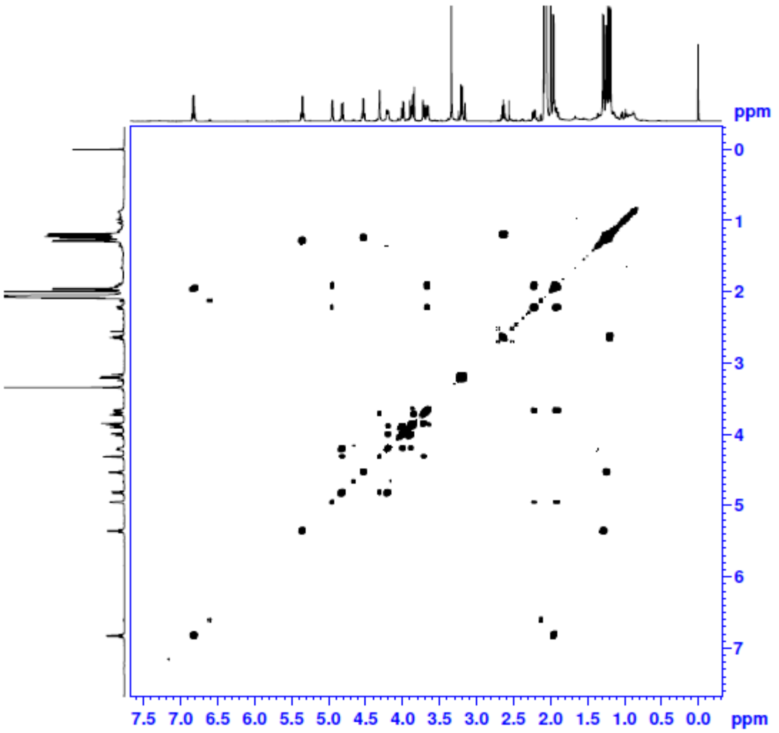

(F)

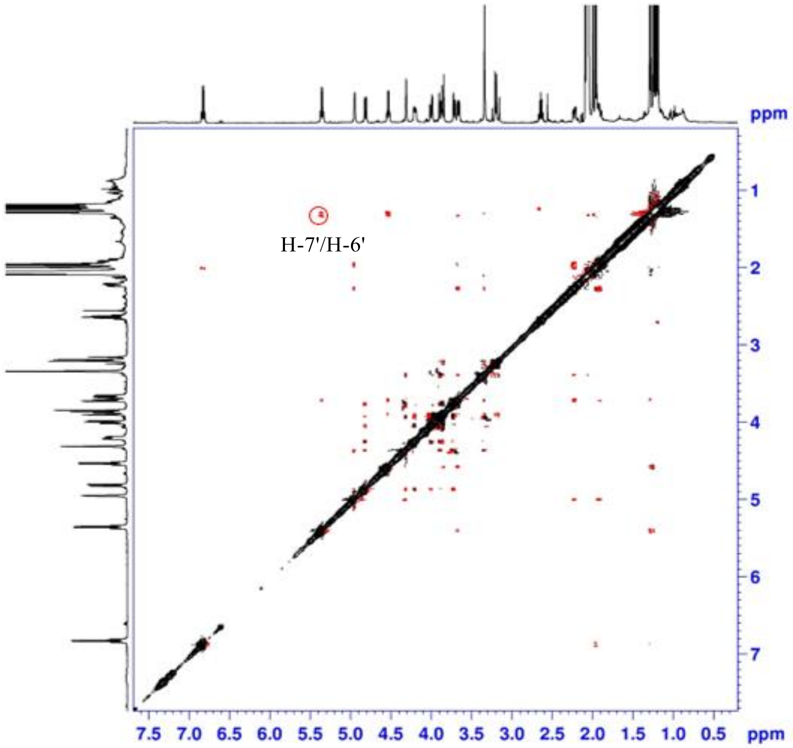

Figure S2-2. Spectral data of compound **4**. (E) ^{1}H-^{1}H COSY spectrum of **4**. (F) NOE spectrum of **4**. The key NOE signal is marked with red circle.

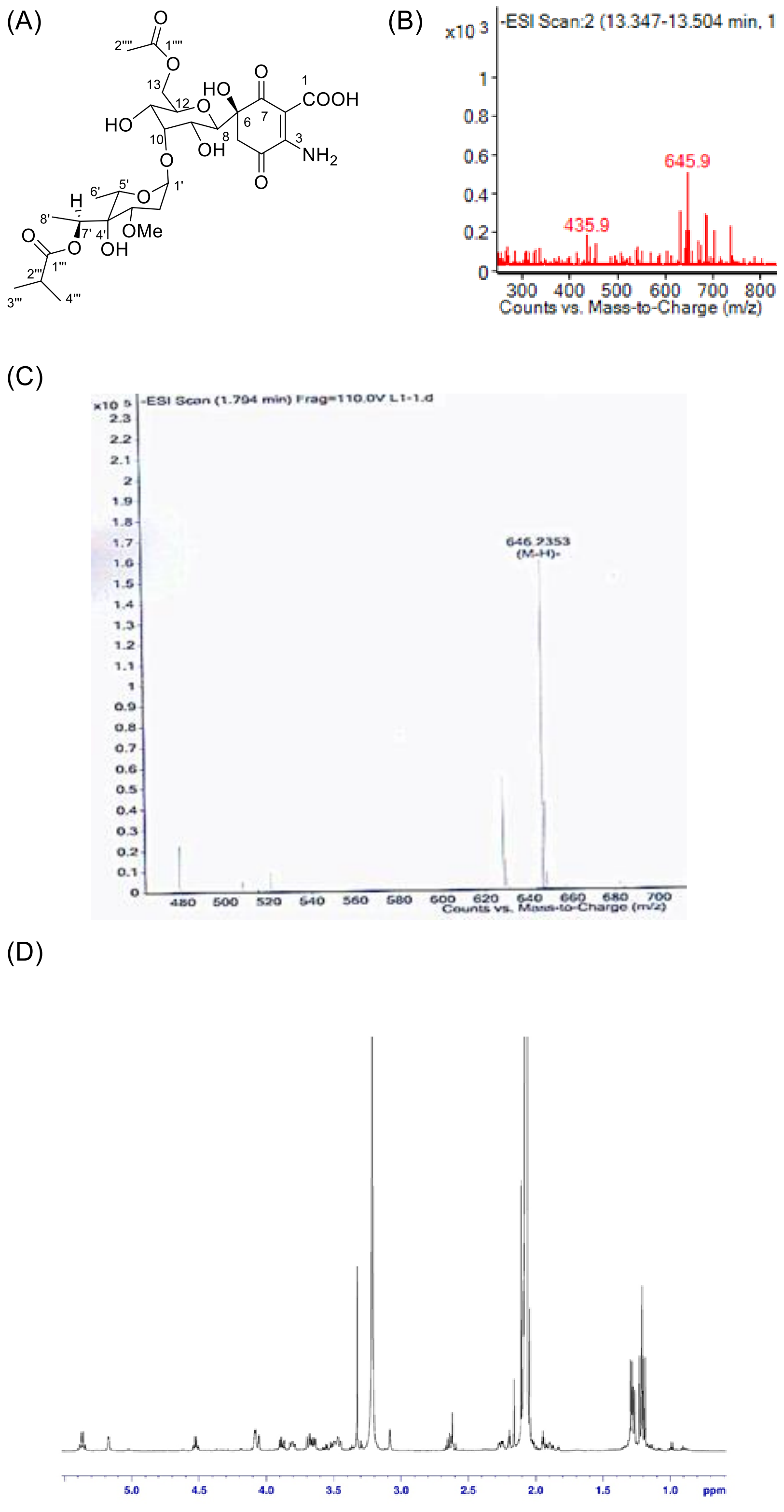

Figure S3-1. Spectral data of compound **6**. (A) Structure of compound **6**. (B) ESI-MS spectrum of **6**. (C) HR-ESI-MS spectrum of **6**. (D) ^{1}H NMR spectrum of **6**.

(E)

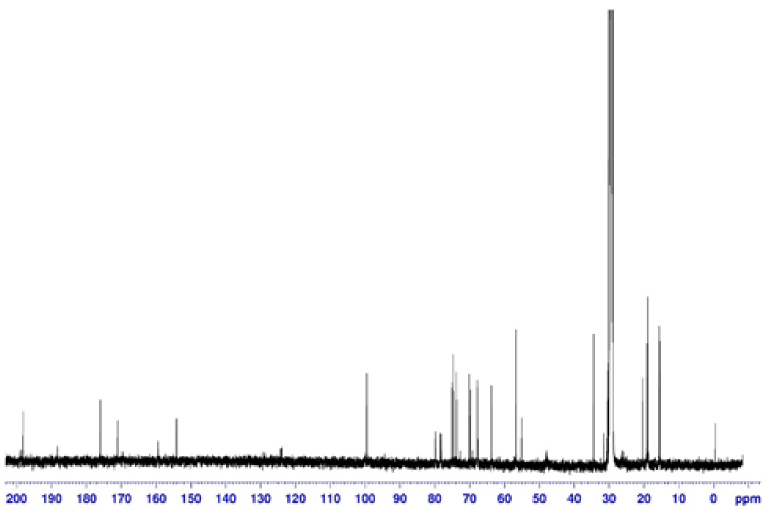

(F)

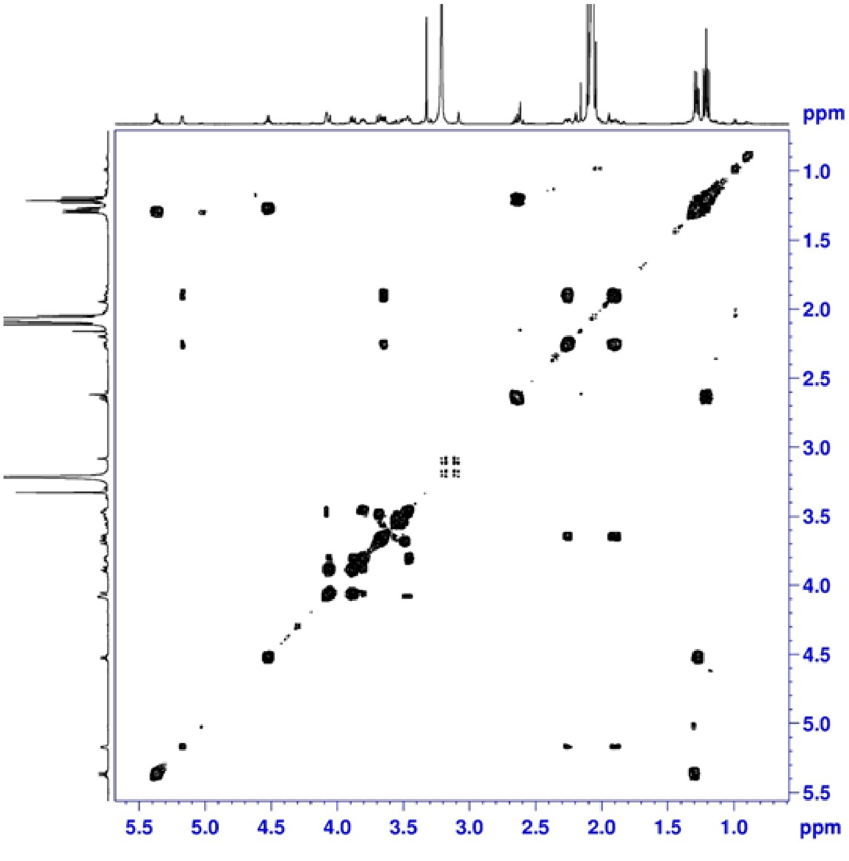

Figure S3-2. Spectral data of compound **6**. (E) ^{13}C NMR spectrum of **6**. (F) ^{1}H-^{1}H COSY spectrum of **6**.

(G)

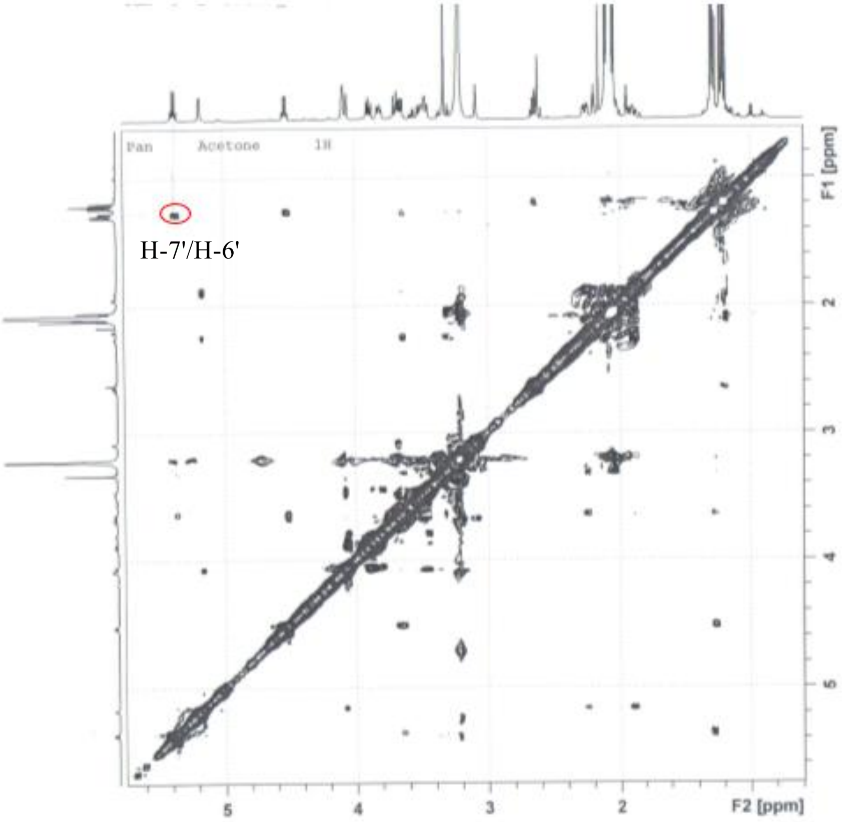

(H)

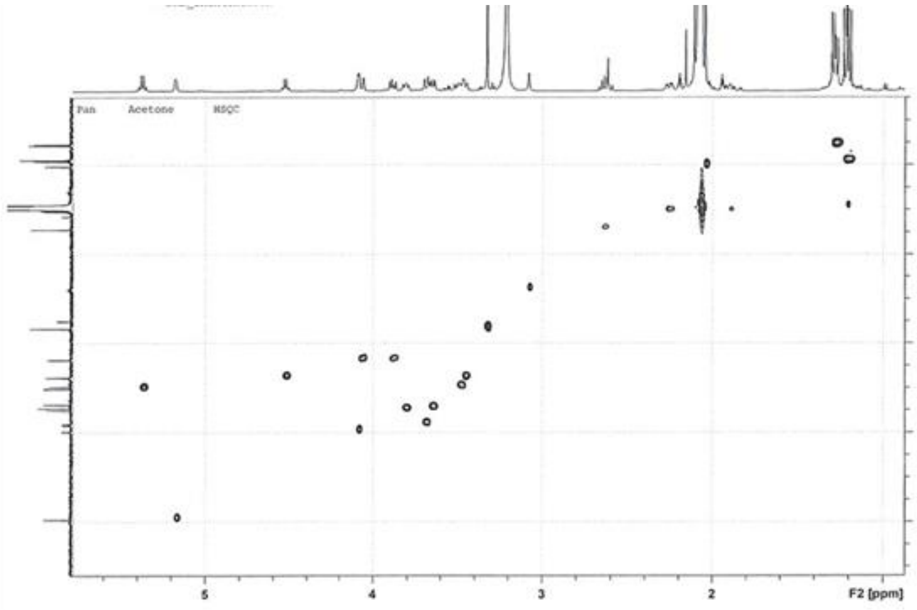

Figure S3-3. Spectral data of compound **6**. (G) NOE spectrum of **6**. The key NOE signal is marked at the spectrum with red circle. (H) HSQC spectrum of **6**.

(I)

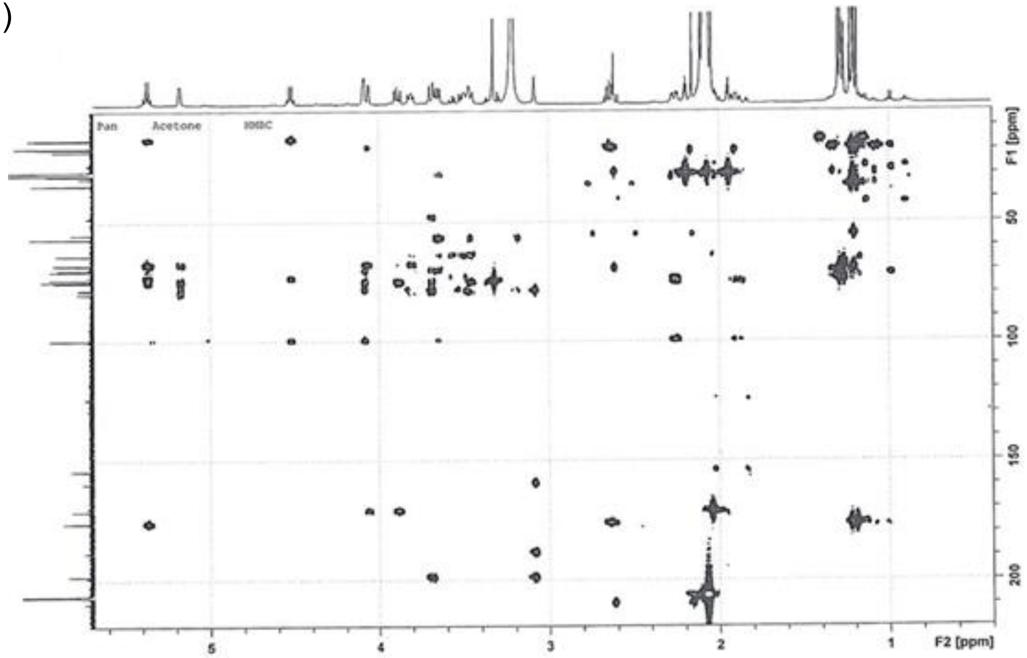

Figure S3-4. Spectral data of compound **6**. (I) HMBC spectrum of **6**.

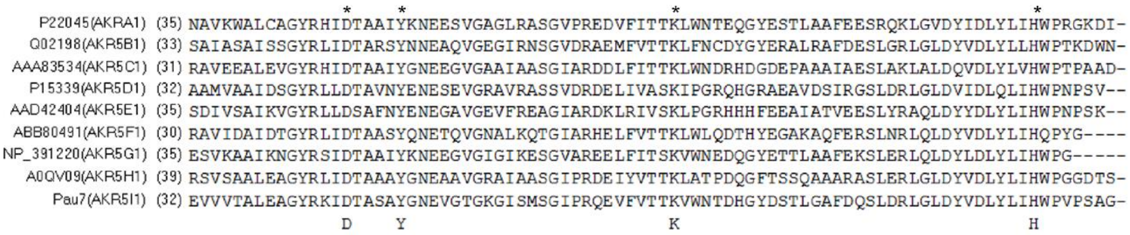

Figure S4. Multiple alignments of Pau7 and aldo-keto reductases (AKRs) belonging to the AKR5 subfamily. The catalytic tetrad residues (D, Y, K and H) are marked with asterisk. P22045 represents AKR5A subfamily; Q02198 represents AKR5B subfamily; AAA83534 represents AKR5C subfamily; P15339 represents AKR5D subfamily; AAD42404 represents AKR5E subfamily; ABB80491 represents AKR5F subfamily; NP_391220 represents AKR5G subfamily; A0QV09 represents AKR5H subfamily.

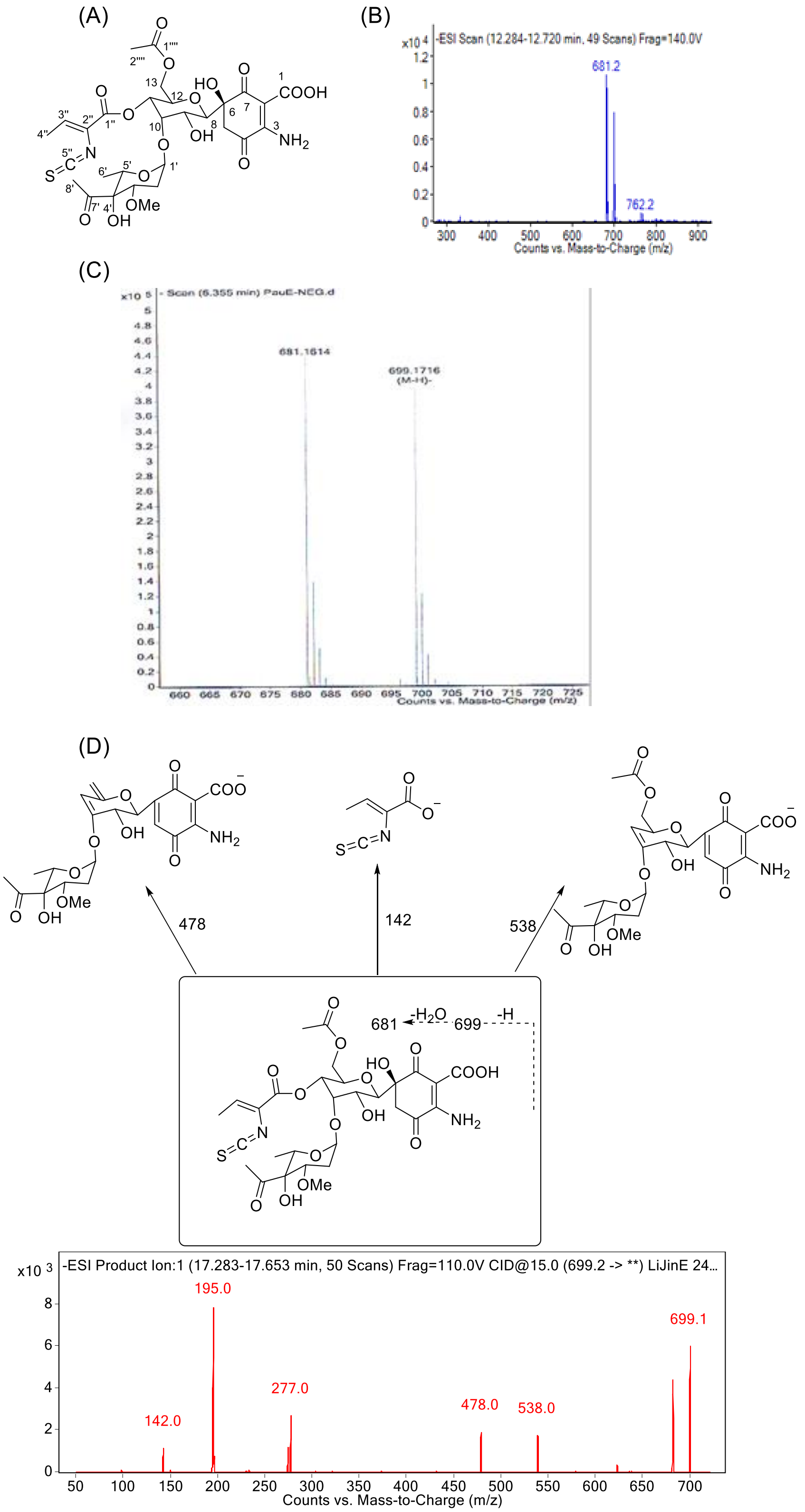

Figure S5. Spectral data of compound **1**. (A) Structure of compound **1**. (B) ESI-MS spectrum of **1**. (C) HR-ESI-MS spectrum of **1**. (D) Tandem MS analysis of **1**.

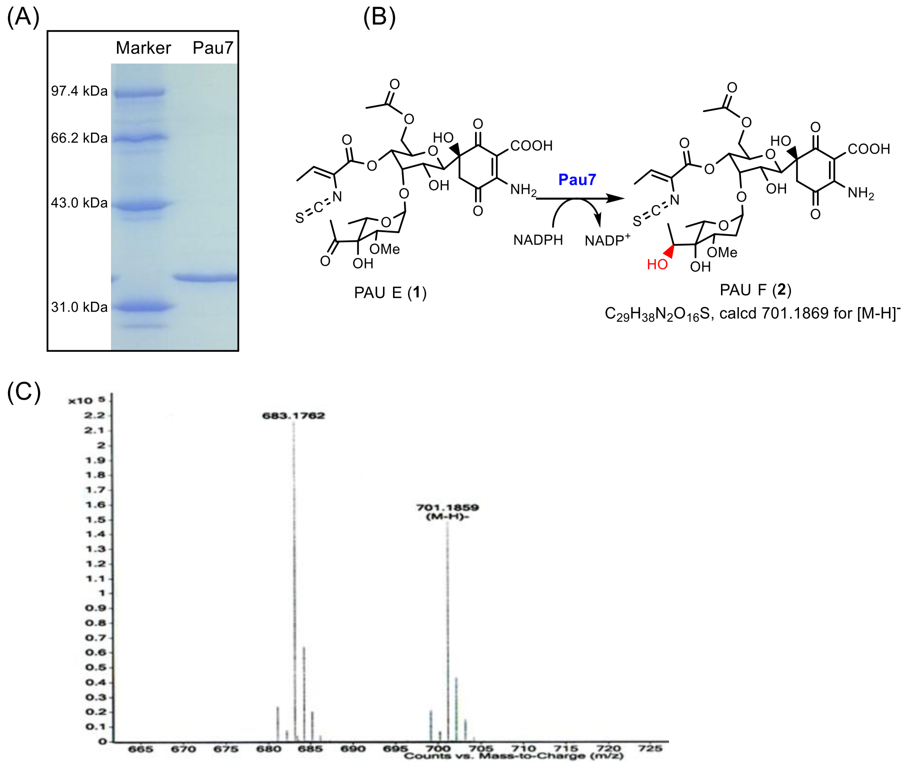

Figure S6. Reaction catalyzed by Pau7. (A) SDS-PAGE analysis of purified N-His6-tagged Pau7. (B) Scheme showing the conversion from **1** to **2** by Pau7. (C) HR-ESI-MS spectrum of the product **2**.

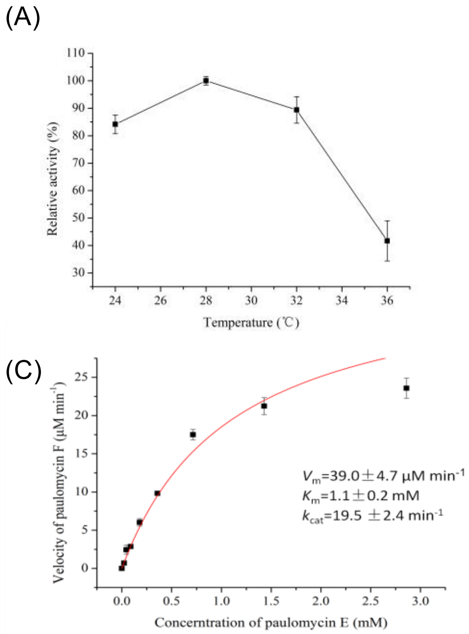

Figure S7. Optimization of Pau7 reaction conditions and steady-state kinetic analysis of Pau7. (A) Optimizing temperatures of Pau7 assay. (B) Optimizing pH values of Pau7 assay. (C) Steady-state kinetic analysis of Pau7. Determination of the kinetic parameters for PAU E (**1**) with saturating NADPH (4 mM).

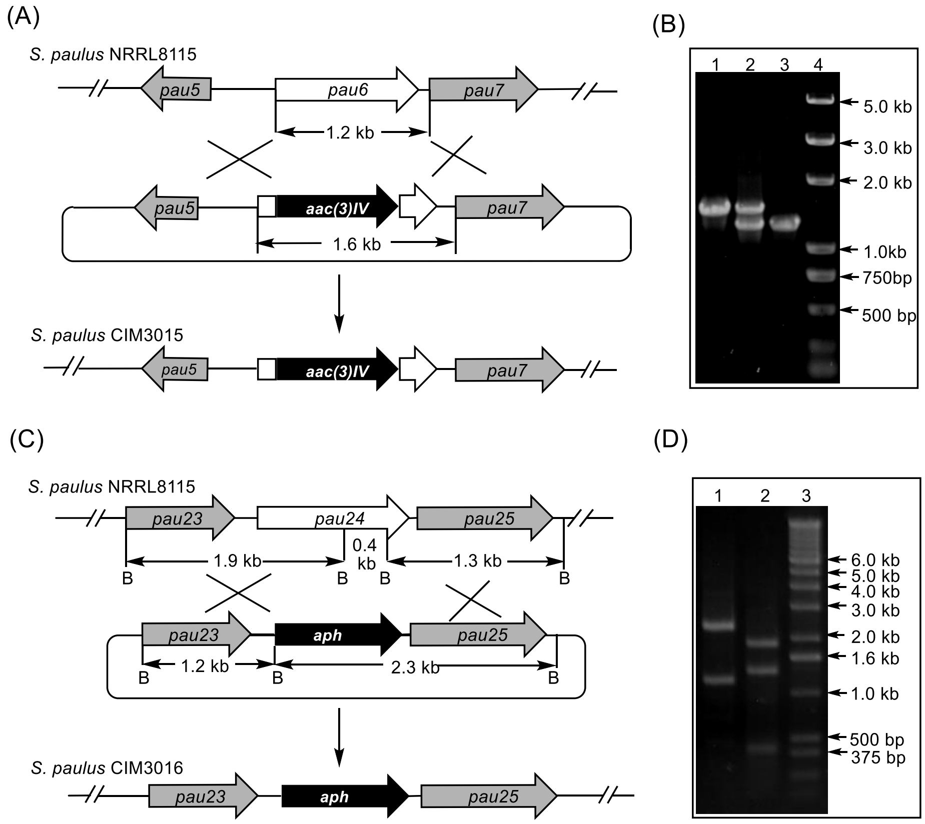

Figure S8. Construction of the Δ*pau6* mutant CIM3015 and Δ*pau24* mutant CIM3016. (A) Diagram illustrating the construction of CIM3015 by replacing *pau6* with an apramycin-resistance gene cassette (*aac(3)IV*). (B) PCR detection of *pau6* inactivation. Lane 1, fragments obtained by PCR using CIM3015 as a template; lane 2, fragments obtained by PCR using a single-cross mutant as a template; lane 3, fragments obtained by PCR using *S. paulus* NRRL 8115 as a template; Lane 4, DNA Ladder. (C) Diagram illustrating the construction of CIM3016 by replacing *pau24* using a kanamycin-resistance gene cassette (*aph*). (D) PCR detection of *pau24* inactivation. Lane 1, fragments obtained by PCR using CIM3016 as a template and a following *Bam*HI digestion; lane 2, fragments obtained by PCR using *S. paulus* NRRL 8115 as a template and a following *Bam*HI digestion; Lane 3, DNA Ladder. B, *Bam*HI.

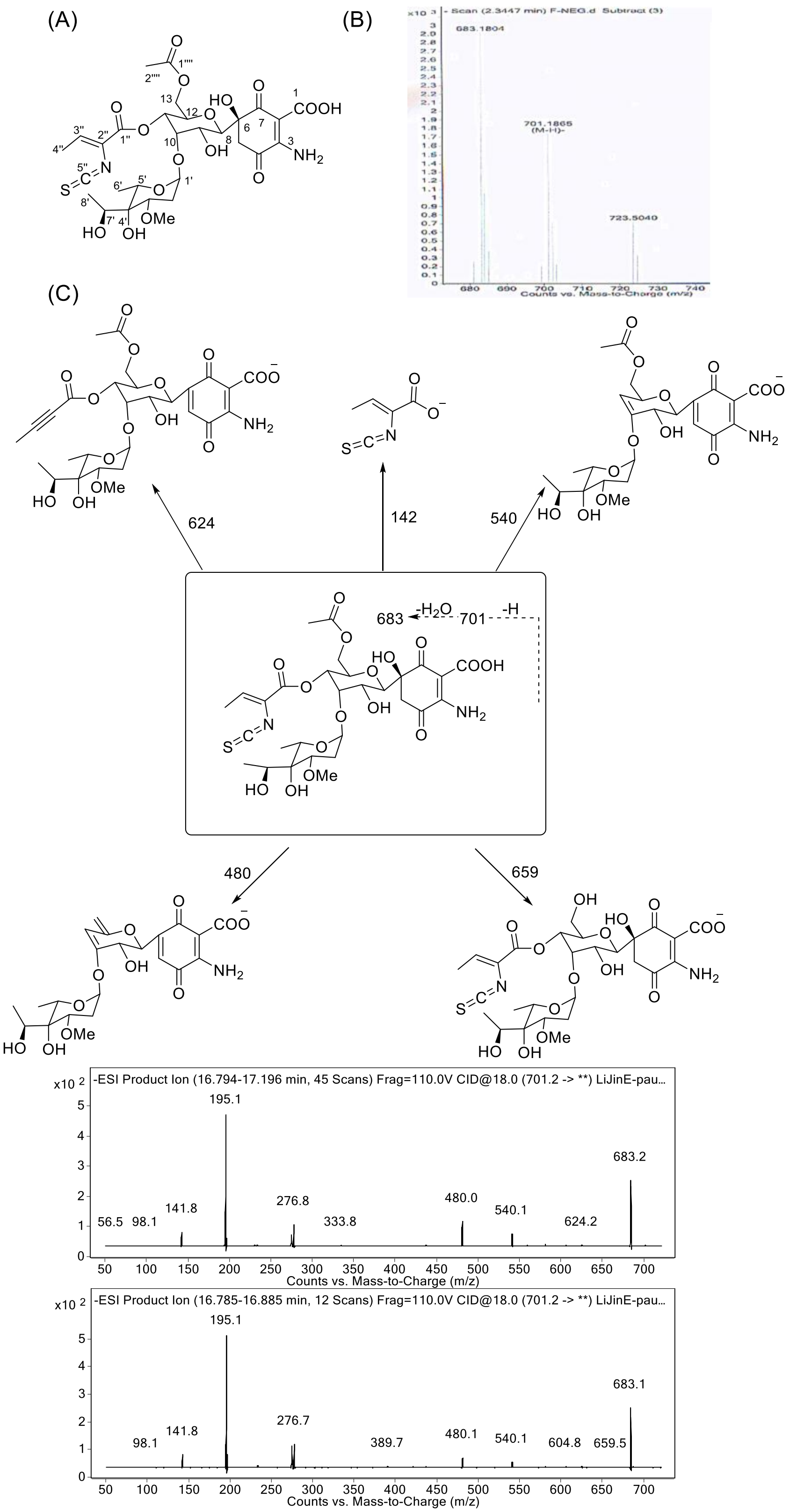

Figure S9. Spectral data of compound **2**. (A) Structure of compound **2**. (B) HR-ESI-MS spectrum of **2**. (C) Tandem MS analysis of **2**.

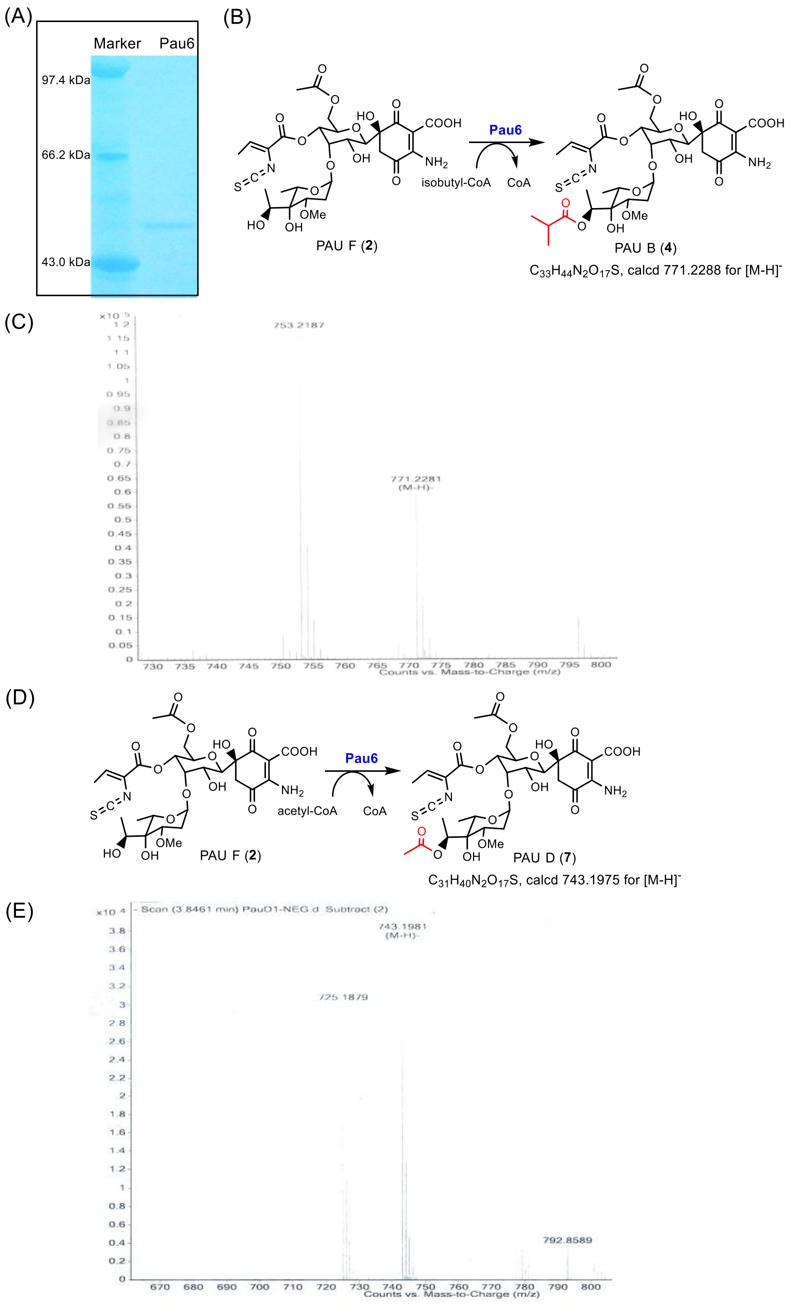

Figure S10. Reaction catalyzed by Pau6. (A) SDS-PAGE analysis of purified N-His6-tagged Pau6. (B) Scheme showing the conversion from **2** to **4** by Pau6. (C) HR-ESI-MS spectrum of the product **4**. (D) Scheme showing the conversion from **2** to **7** by Pau6. (E) HR-ESI-MS spectrum of the product **7**.

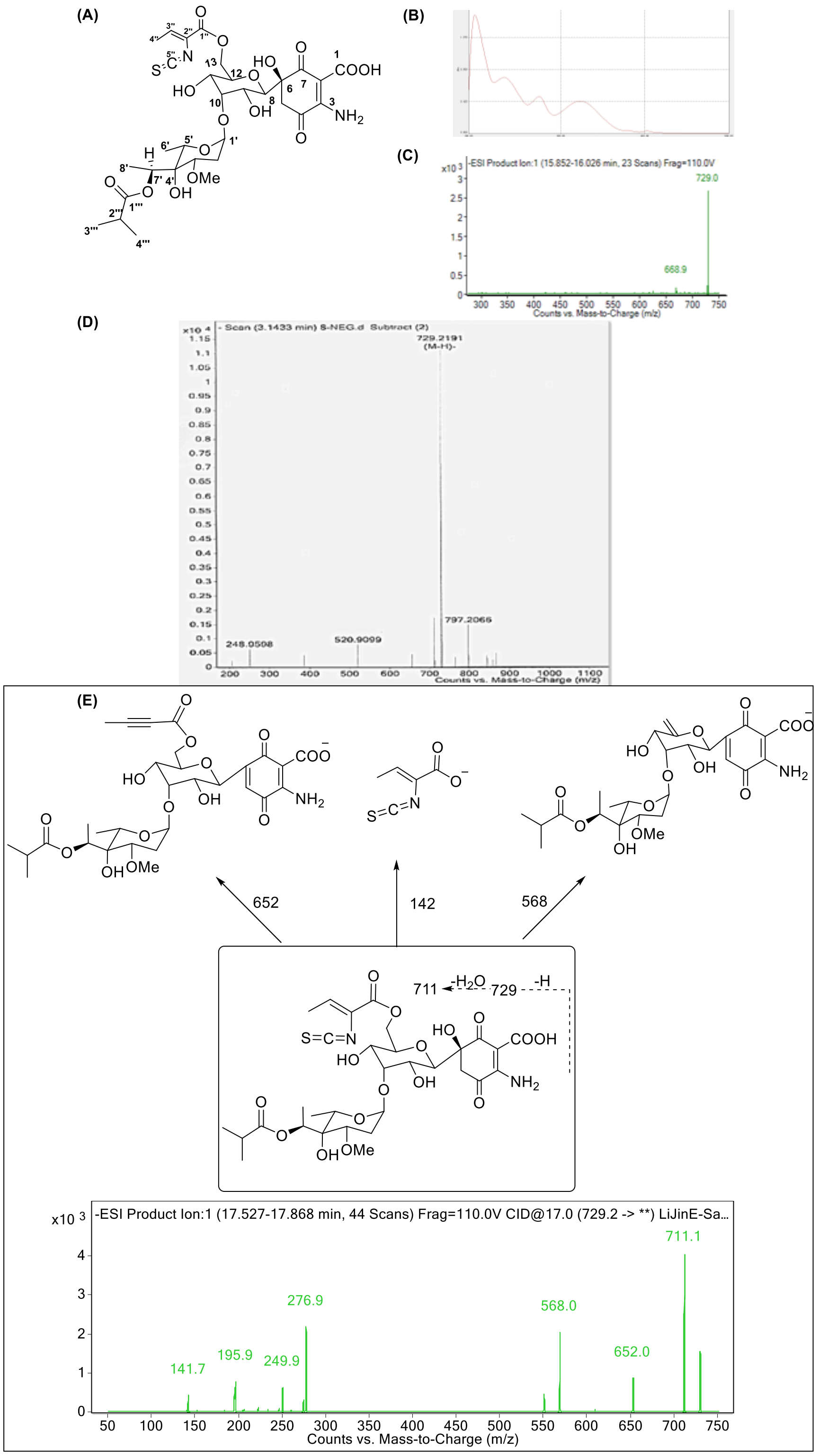

Figure S11-1. Spectral data of compound **8**. (A) Structure of compound **8**. (B) UV spectrum (95 μM in acetonitrile) of **8.** (C) ESI-MS spectrum of **8**. (D) HR-ESI-MS spectrum of **8**. (E) Tandem MS analysis of **8**.

(F)

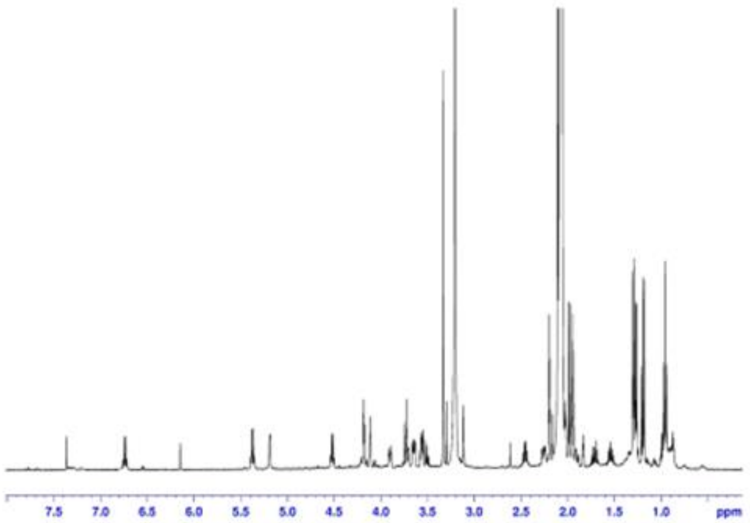

(G)

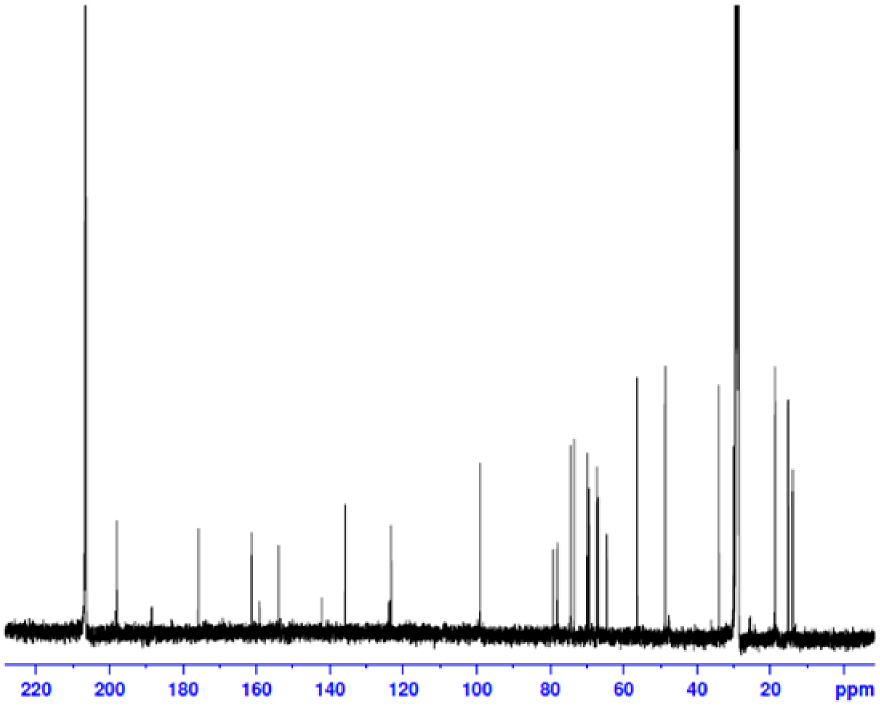

Figure S11-2. Spectral data of compound **8**. (F) ^{1}H NMR spectrum of **8**. (G) ^{13}C NMR spectrum of **8**.

(H)

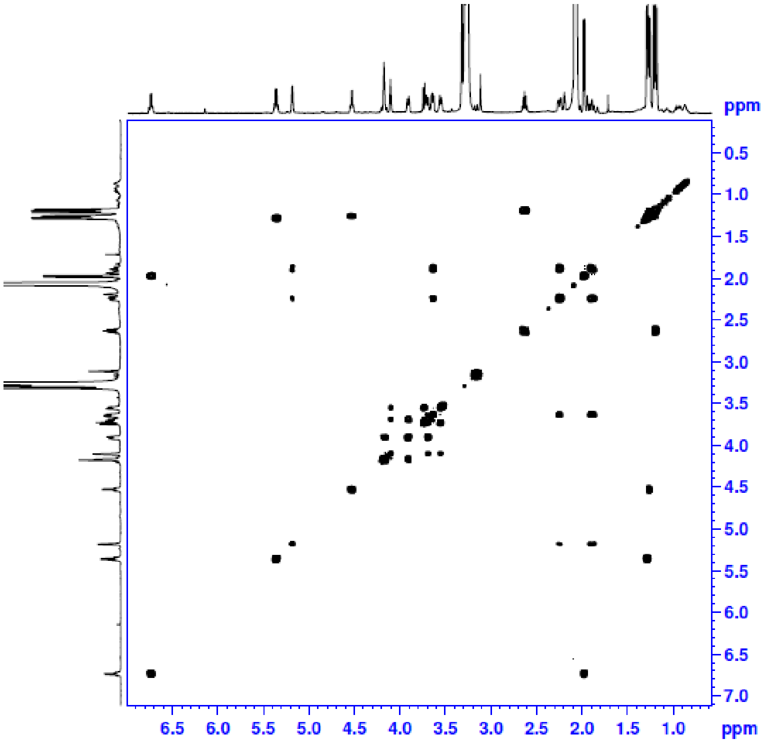

(I)

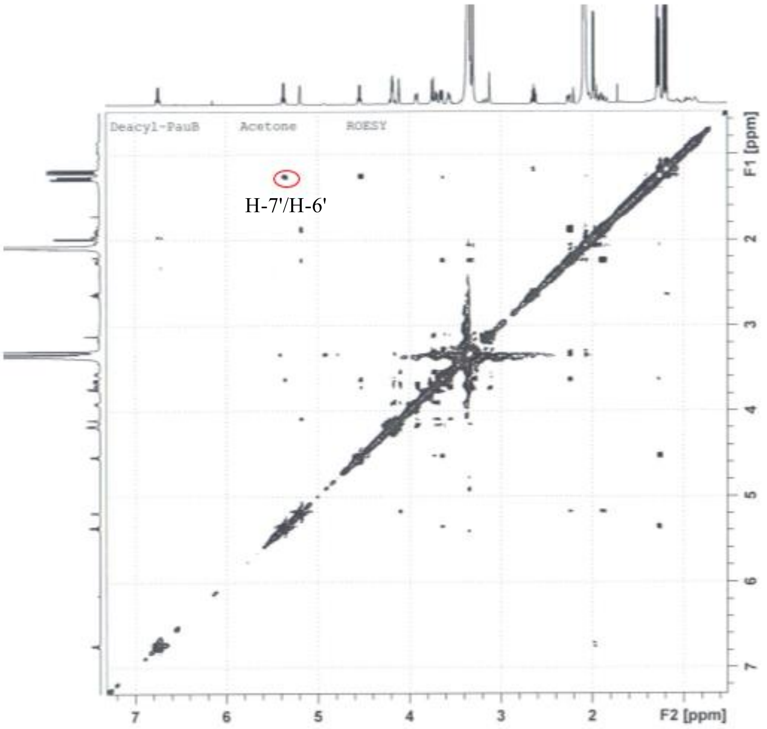

Figure S11-3. Spectral data of compound **8**. (H) ^{1}H-^{1}H COSY spectrum of **8**. (I) NOE spectrum of **8**. The key NOE signal is marked with red circle.

(J)

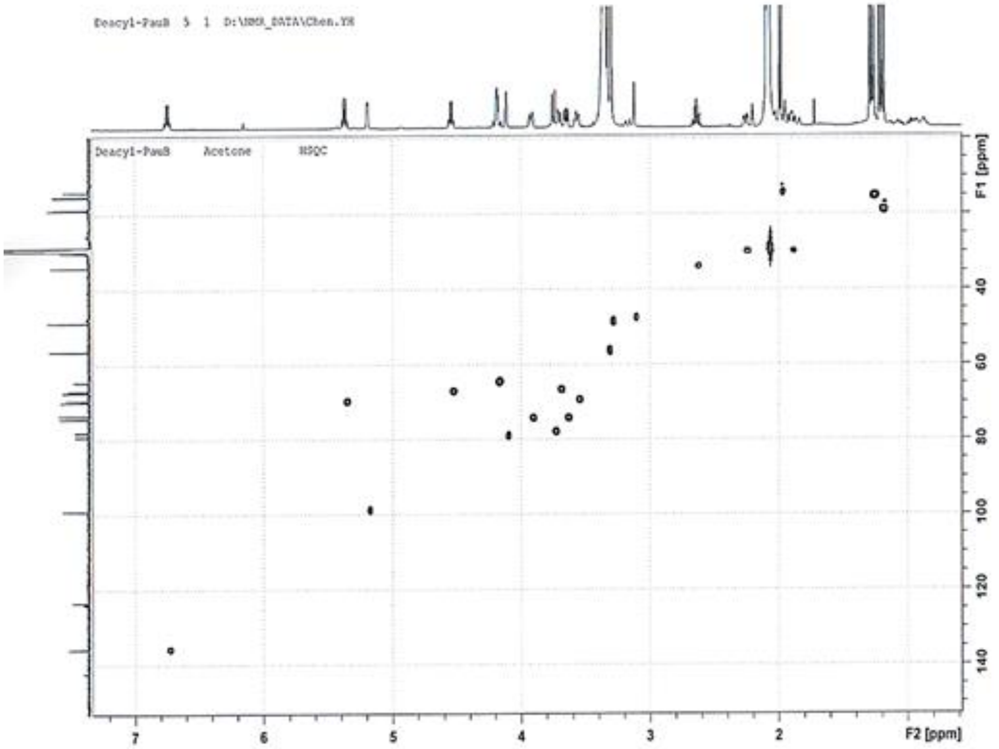

(K)

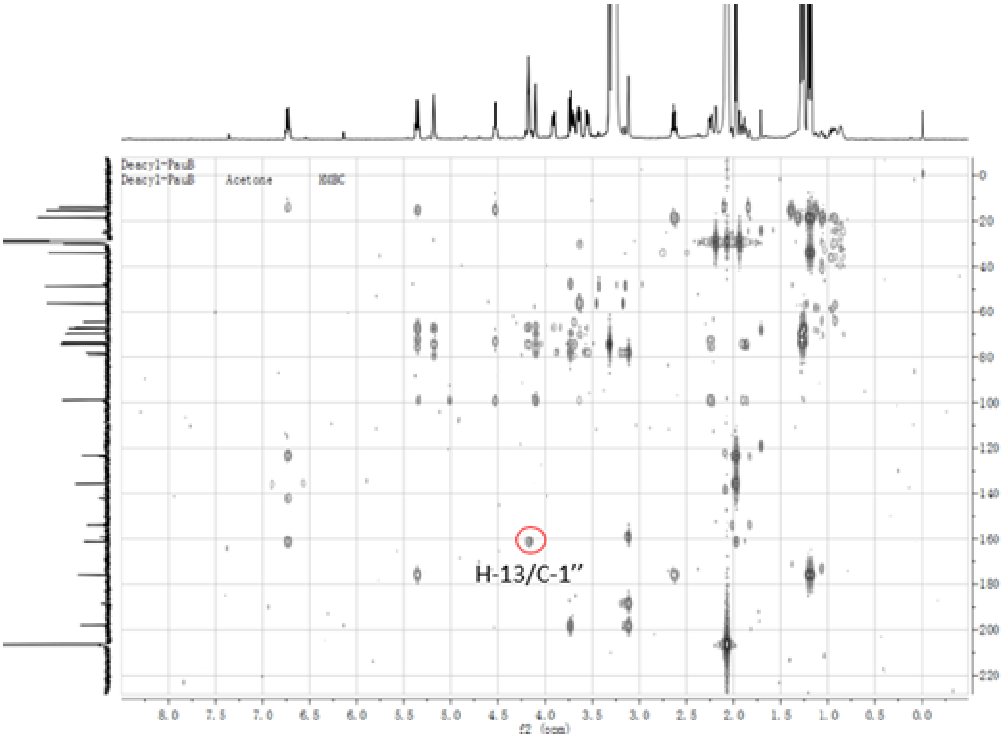

Figure S11-4. Spectral data of compound **8**. (J) HSQC spectrum of **8**. (K) HMBC spectrum of **8**. The key HMBC signal is marked with red circle.

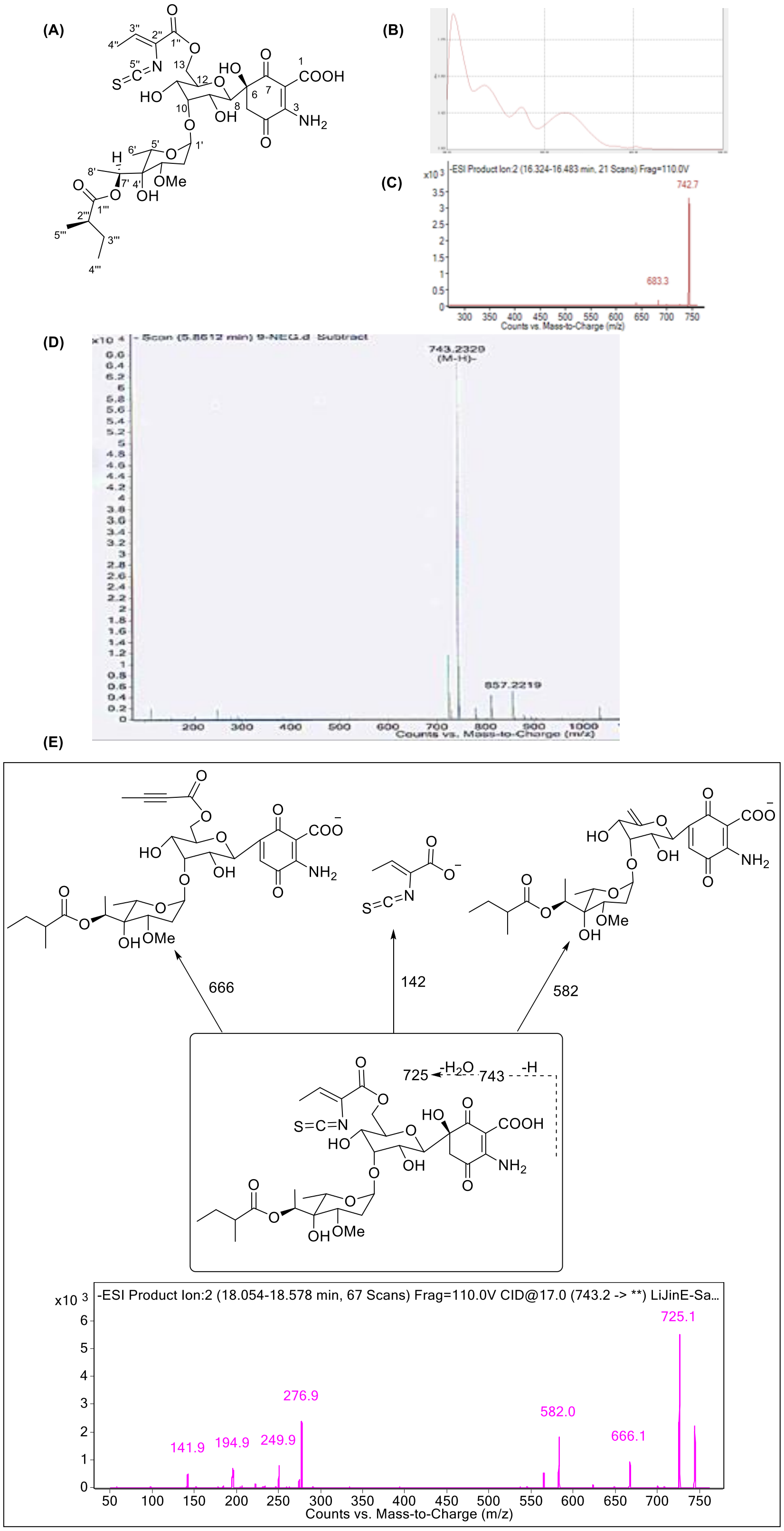

Figure S12-1. Spectral data of compound **9**. (A) Structure of compound **9**. (B) UV spectrum of **9** (48 μM in acetonitrile). (C) ESI-MS spectrum of **9**. (D)HR-ESI-MS spectrum of **9**. (E) Tandem MS analysis of **9**.

(F)

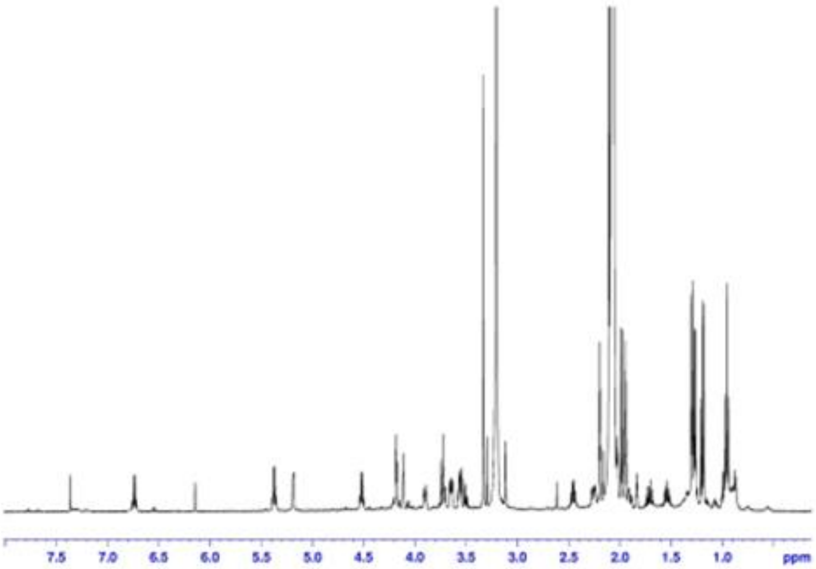

(G)

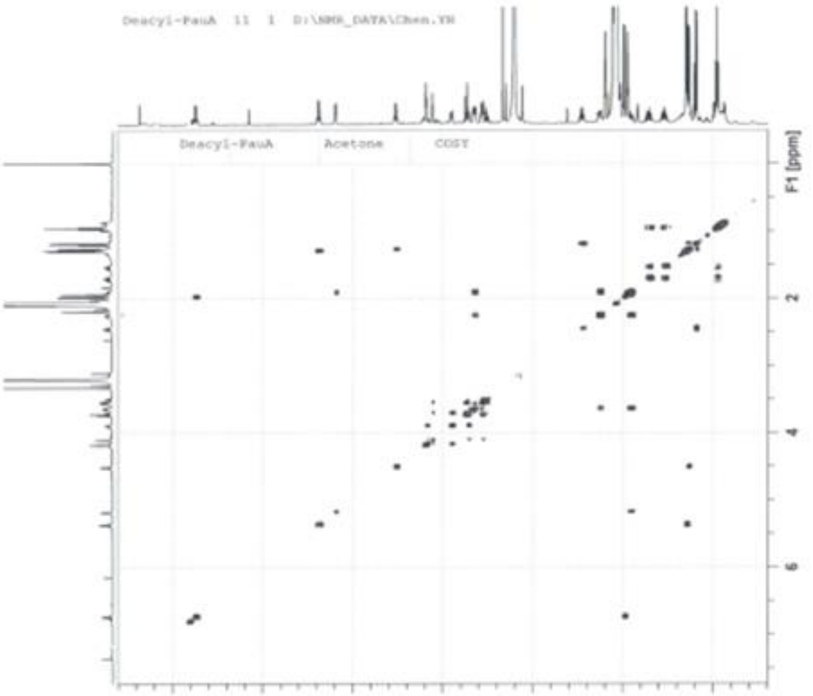

Figure S12-2. Spectral data of compound **9**. (F) ^{1}H NMR spectrum of **9**. (G) ^{1}H-^{1}H COSY spectrum of **9**.

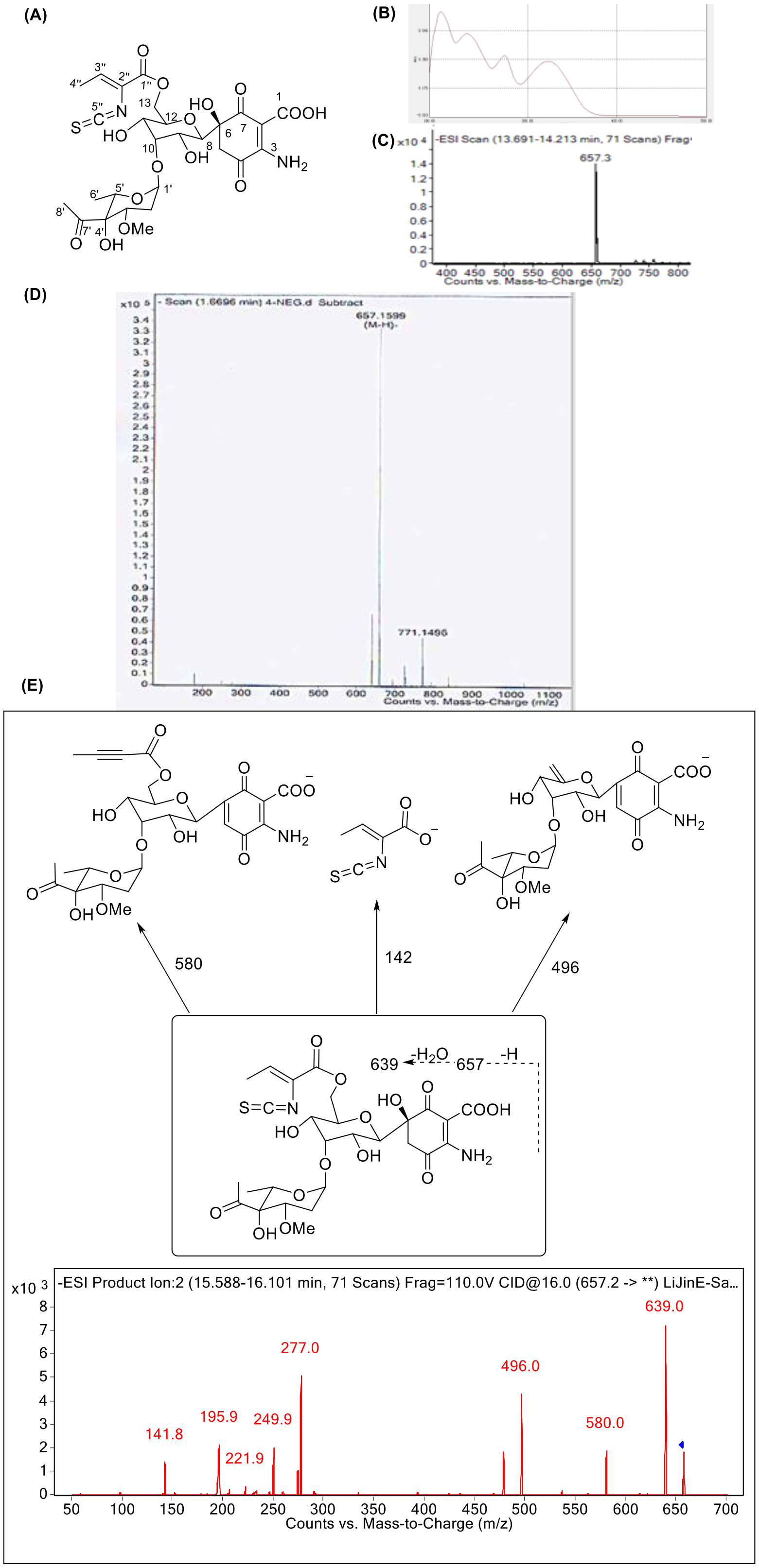

Figure S13-1. Spectral data of compound **10**. (A) Structure of compound **10**. (B) UV spectrum of **10** (106 µM in acetonitrile). (C) ESI-MS spectrum of **10**. (D) HR-ESI-MS spectrum of **10**. (E) Tandem MS analysis of **10**.

(F)

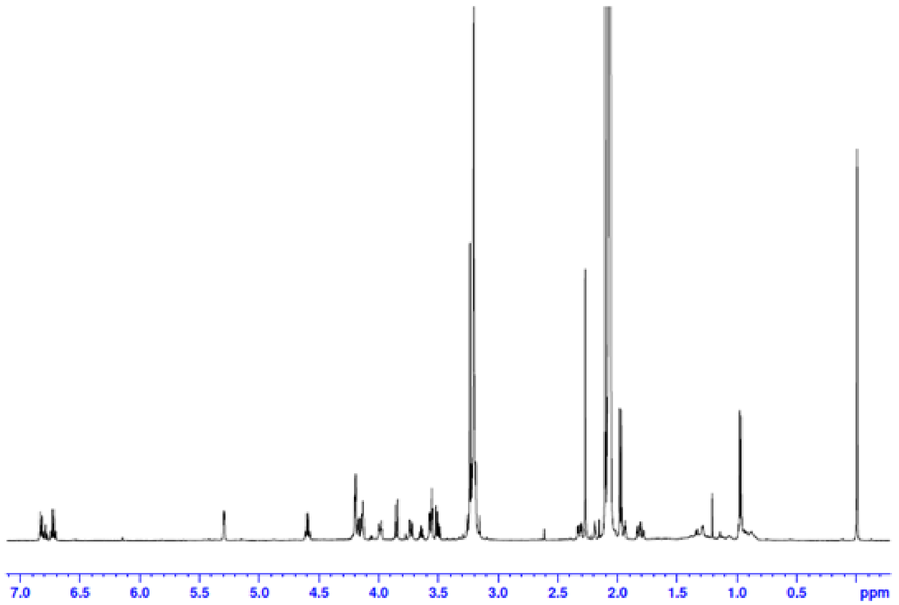

(G)

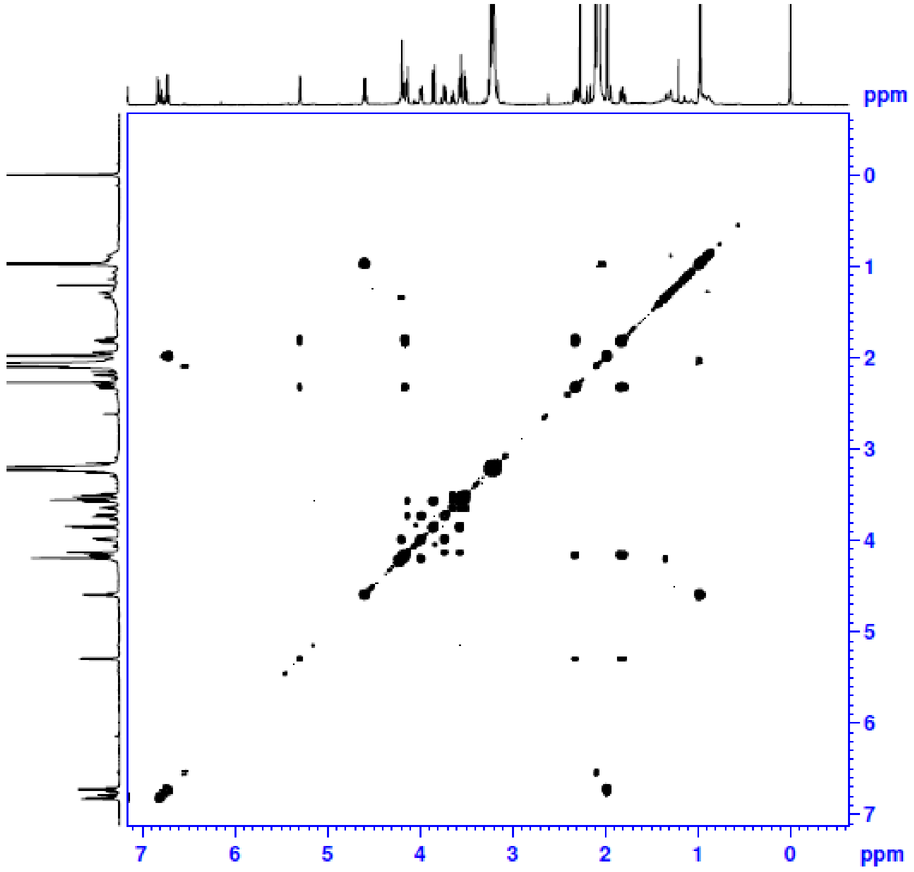

Figure S13-2. Spectral data of compound **10**. (F) ^{1}H NMR spectrum of **10**. (G) ^{1}H-^{1}H COSY spectrum of **10**.

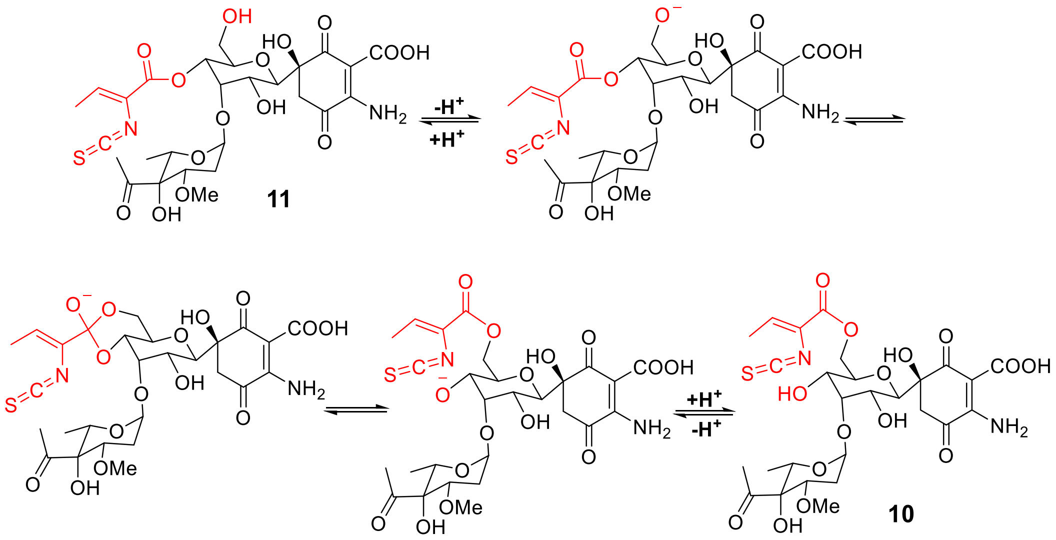

Figure S14. A proposed mechanism of the pauloyl group migration.
